# Recent Progress on Fluorescent Probes in Heavy Metal Determinations for Food Safety: A Review

**DOI:** 10.3390/molecules28155689

**Published:** 2023-07-27

**Authors:** Liqing Lai, Fang Yan, Geng Chen, Yiwen Huang, Luqiang Huang, Daliang Li

**Affiliations:** 1 The Public Service Platform for Industrialization Development Technology of Marine Biological Medicine and Products of the State Oceanic Administration, Fujian Key Laboratory of Special Marine Bioresource Sustainable Utilization, College of Life Sciences, Fujian Normal University, Fuzhou 350117, China; lqinglai7@163.com (L.L.); yanfang8270@163.com (F.Y.); 2 Fujian Fishery Resources Monitoring Center, Fuzhou 350117, China; cg395395@126.com (G.C.); m15259109300@163.com (Y.H.)

**Keywords:** fluorescent probes, heavy metal detection, food safety

## Abstract

One of the main challenges faced in food safety is the accumulation of toxic heavy metals from environmental sources, which can sequentially endanger human health when they are consumed. It is invaluable to establish a practical assay for the determination of heavy metals for food safety. Among the current detection methods, technology based on fluorescent probes, with the advantages of sensitivity, convenience, accuracy, cost, and reliability, has recently shown pluralistic applications in the food industry, which is significant to ensure food safety. Hence, this review systematically presents the recent progress on novel fluorescent probes in determining heavy metals for food safety over the past five years, according to fluorophores and newly emerging sensing cores, which could contribute to broadening the prospects of fluorescent materials and establishing more practical assays for heavy metal determinations.

## 1. Introduction

Metal elements are widely used in our daily lives by virtue of their good thermal and electrical conductivities, special luster, and excellent ductility. However, with the rapid development of the modern economy, excessive discharge of waste from industry, random abuse of metal-containing pesticides and fertilizers, and improper handling of common daily necessities containing metals have led to the massive discharge of heavy metals as ions into the soil and surface water [1,2]. Heavy metals in the environment can be easily accumulated in aquatic products, crops, and other foodstuffs in the form of ions and then be absorbed into the human body through the food chain, which gradually increases the threat to health via unconscious intaking of heavy metals in our daily lives [3,4].

Typically, heavy metals are considered as inorganic irritant toxicants and are generally classified into three groups: toxic metals (e.g., mercury, lead, chromium, cadmium, nickel, arsenic, cobalt, tin), precious metals (e.g., palladium, platinum, silver, gold, plutonium), and radionuclides (e.g., uranium, thorium, radium, americium) [5]. In particular, mercury, lead, chromium, and cadmium are present at high levels in the environment and accumulated easily in the food chain [6,7]. It has been shown that these heavy metals are prone to react with biomolecular affinity sites and trigger structural changes in biological function molecules in living organisms due to their well-coordinated interactions with biological function molecules containing nitrogen (N), oxygen (O), and sulfur (S), thus affecting various cellular enzymes and protein systems inside the body and disrupting their normal physiological effects [8,9]. For example, mercury (Hg) induces oxidative damage to the mucosa of the gastrointestinal tract and proximal renal tubules, which manifests clinically as abdominal pain, hemorrhagic gastroenteritis, acute tubular necrosis, and subacute shock [10,11]. The pathophysiological toxicity of lead (Pb) is fairly complicated as it involves almost every organ system, with the most severe neurological manifestations being seizures and coma [12,13,14]. Cadmium (Cd) can disrupt iron homeostasis in humans by inducing hyperactivation of heme oxygenase-1 (HO-1) and disrupting lipid metabolism, which ultimately leads to iron apoptosis [15,16,17,18]. The epidemiology of infertility has been shown to be related to the impact of exposure to the heavy metals lead and cadmium [19,20]. Chromium (Cr)-induced ROS-mediated oxidative stress has been disclosed to lead to redox imbalance and affect the balance of antioxidant systems in the body [21]. In conclusion, heavy metals can be uptaken through the respiratory, digestive, and dermal tracts, enter the bloodstream, and rapidly distribute to organs and tissues throughout the body, which ultimately leads to neurological disorders, skin and vascular damage, immune system dysfunction, gastrointestinal and renal dysfunction, and even some cancers. Therefore, the development of rapid, sensitive, and specific-response qualitative and quantitative methods for the detection of heavy metals in food and agriculture-related matrices is of great importance for food safety, environmental monitoring, and clinical diagnosis.

**Scheme 1:**
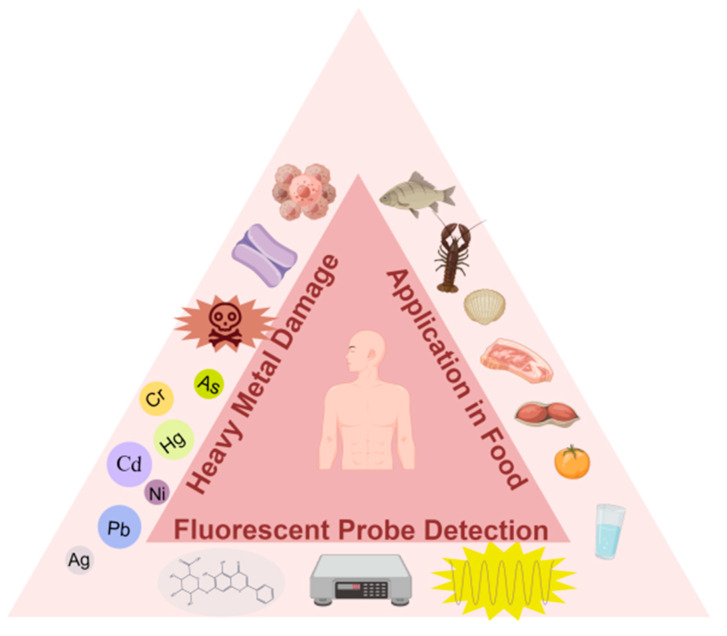
Fluorescent probe-based assays for determining heavy metals in food (e.g., Cr, Cd, Hg, Pb, Ag, As).

The determination of heavy metals is facilitated by applying novel instrumental methods, such as atomic absorption spectrometry, neutron activation analysis, X-ray fluorescence spectrometry, ion chromatography, Raman spectroscopy, etc., toward the detection of metal elements [22,23,24]. Among them, fluorescent small molecule materials have attracted much attention because of their short response time, simple operation, high selectivity and sensitivity, and greater suitability for the analysis of trace metal ions in complex matrices [25,26] (Scheme 1). Therefore, this review comprehensively presents the recent research progress on optical techniques for the detection of heavy metal ions in food over the past five years, especially in terms of analytical methods based on fluorescent materials, attempting to provide a deeper understanding of the challenges and future prospects of their applications.

## 2. Common Fluorescence Spectroscopy Detection Methods

Rapid developments in photochemistry have greatly facilitated the development of instruments and methods for the convenient and accurate detection of metals, quasi-metals, and selected non-metallic elements at (super) trace levels since the 1980s [27]. Common spectroscopic analytical methods include inductively coupled plasma emission spectrometry (**ICP-MS**), atomic fluorescence spectrometry (**AFS**), atomic absorption spectrometry (**AAS**), atomic emission spectrometry (**AES**), X-ray fluorescence spectrometry (**XRF**), etc. [22,28]. Further breakthroughs in convenience and sensitivity have been achieved by simplifying sample pre-treatment, combining different analytical methods in recent years, and optimizing analytical conditions, thereby enabling the monitoring of the spatial distribution of heavy metals in food entities [29] (Table 1).

**ICP-MS** has been reported in the literature as the most effective quantitative analytical method for measuring trace elements in food samples (such as peanuts [30], salted foods, and sea salt [31]), due to its high sensitivity and selectivity. Additionally, it can be combined with other technologies for superior detection efficiency. A new method for the efficient monitoring of the spatial distribution of Hg and Se in mushrooms (with a spatial resolution as low as 5 µm) was proposed for the first time, with researchers using laser ablation (**LA**) and **ICP-MS** to quantify mercury and selenium in mushroom substrates with detection limits of 0.006 and 0.3 µg·g^−1^, respectively [32]. For accomplishing the simultaneous determination of trace selenium and cadmium in rice samples, G. Lan et al. [33] first introduced bypass gas to modify graphite furnace electrothermal evaporation (**GF-ETV**) and **ICP-MS**. Through optimization, the detection limits of selenium and cadmium were as low as 0.5 and 0.16 µg·kg^−1^ respectively, with repeated determinations exhibiting relative standard deviations (RSDs) within 8% (*n* = 6). Pre-concentration of samples to enhance sensitivity is another focus of improvement schemes. Solid phase extraction (**SPE**) is the simplest method for pre-concentration. Compared with the classical liquid–liquid extraction method, **SPE** presents advantages such as less sample consumption, higher multiples of accumulation, better recoveries, rapid phase separation, cheaper costs, etc. Based on these advantages, a method was constructed with flow injection (**FI**) and **SPE-ICP-MS** [34]. It combined chemometric methods with experimental design and multi-response surface methodology to simultaneously determine toxic elements. The method successfully reduced the detection limits, ranging from 0.8 ng·L^−1^ for Mn to 0.09 μg·L^−1^ for Hg, with relative standard deviations all < 5%, and was successfully applied to the determination of heavy metals in rice and rice products. Similarly, D. Chen et al. [35] established a simple and efficient method, which combined high-performance liquid chromatography–atomic fluorescence spectrometry (**HPLC-AFS**) and **SPE**. The method showed high sorption capacity and a wide range of adaptability for the simultaneous determination of four forms of mercury (Hg^2+^, MeHg, EtHg, PhHg). Under optimized conditions, the limits of detection were 0.001–0.002 µg·L^−1^, the recoveries were 87.2–111%, and the reproducibilities were 1.1–6.5% in water samples. Pre-treatment of complex samples before detection is another significant scheme to enhance flexibility and sensitivity. For example, pre-emptive microwave digestion of samples, such as beverages and chocolate, utilizing 20% nitric acid (*v*/*v*) made it possible to decrease the detection limit to parts per trillion (ng·L^−1^) using an **ICP-MS** technique [36]. Similarly, when studying sample pre-treatment methods, such as type, concentrations, and system ratios of ablation reagents, for the combination of **ICP-MS** and atomic emission spectrometry (**AES**), a method for the simultaneous determination of Ag, As, Bi, Cd, Cr, and other heavy metals in turmeric was developed [37].

**AES**, **AFS**, and **AAS** are complementary technologies. They have become mainstream methods for determining heavy metals in agriculture and potable water due to their advantages of low detection limits, high accuracy, good selectivity, less sample consumption, and a wide range of applications. They are suitable for the analysis of trace components in samples such as wheat flour [38], honey [39], and milk powder [40]. Two serially-connected graphite tubes were innovatively applied as two electrolytic cells to form an electrochemical vapor generation atomic fluorescence spectrometry (**EcHG**) system with **AES** [41]. It worked well in the determination of trace Cd without ion exchange membranes, with easy assembly, direct sample detection, and stable signal retention. The limit of detection was 0.05 ng·mL^−1^, with a relative standard deviation of 3.2%, and the **EcHG** efficiency was 38.4 ± 2.2%. Also, it was successfully applied to the determination of potable water samples with recoveries of 95–109%. Coupling with other technologies facilitates further improvements in detection efficiency. A chemical vapor generation multi-channel non-dispersive atomic fluorescence spectrometer (**CVG-NDAFS**) was developed to simultaneously detect arsenic, antimony, selenium, and mercury in herbal foods and biological standards [42]. The optimized method reduced the detection limits to 0.051, 0.034, 0.050, and 0.0058 ng·mL^−1^, respectively, with relative standard deviations of 0.42%, 0.74%, 0.97% and 1.0%, respectively. Solid injection electrothermal evaporation atomic absorption spectrometry (**SS-ETV**) was combined with **AAS** to determine the cadmium content in chocolate [43]. By optimizing the experimental conditions, the limit of detection was successfully decreased from 150 to 70 pg·g^−1^, accompanied by a high correlation coefficient (R^2^ > 0.999). Furthermore, the relative standard deviation of the actual sample detection ranged from 1.5% to 6.4%, indicating a satisfactory level of precision.

**XRF** is an analytical technique that utilizes the absorption variations in samples of X-rays to determine its composition. It offers several advantages, such as rapid analysis, the ability to analyze a wide range of elements, strong applicability, minimal spectral interference, and a nondestructive nature towards the sample. This method is not only capable of analyzing solid block samples but also provides the means to analyze the composition and thickness of individual layers within multilayer coatings [44]. The majority of detection limits can reach up to 10^−6^, and when combined with separation and enrichment techniques, this limit can be further enhanced to 10^−8^. Total reflection X-ray fluorescence analysis (**TXRF**) was successfully applied in the analysis of heavy metal components in herbal medicines [45]. Based on high definition X-ray fluorescence spectroscopy (**HDXRF**), a method for the rapid quantification of arsenic (As), cadmium (Cd), nickel (Ni), lead (Pb), tin (Sn), and zinc (Zn) in scallops was developed [46], with detection limits of 0.072, 0.070, 0.502, 0.063, 0.033, and 4.383 mg·kg^−1^, respectively. The RSD values of precision, reproducibility, and stability assays were found to be less than 10%. In recent years, portable instruments have been successfully developed to further broaden the practical applicability of **XRF**. G. E. Acquah et al. [47] reported a portable handheld X-ray fluorescence (**pXRF**) spectrometer to successfully achieve highly accurate and efficient detection of trace heavy metal contaminants (chromium, nickel, and arsenic) in fertilizers. Researchers evaluated the common **pXRF** method, acid ablation inductively coupled plasma mass spectrometry, and diphenylcarbonyldihydrazide colorimetric methods for the assessment of lead detection in 69 spices worldwide, which helped to reduce lead exposure [48].

Laser-induced breakdown spectroscopy (**LIBS**) technology has gradually emerged in the technical field of heavy metal detection owing to its rapid detection and green advantages [49]. Q. Zhao et al. [50] brought forth a new idea that constructed a heavy metal content prediction model using near-infrared (NIR) and **LIBS** spectral data, with simultaneous multi-element detection and prediction accuracy as high as 0.90. The coefficients of determination in the optimal prediction models for Zn, Cu, and Pb were 0.9858, 0.9811, and 0.9460, respectively, and the root mean square errors of prediction were 4.3047, 4.9592, and 8.3881 mg·kg^−1^, respectively, which provided good reproducibility for the rapid detection of heavy metals in lilies.

**Table 1:** Common fluorescence spectrometry technologies for the detection of heavy metals in food.

Method	Analytes	LOD	Sample	Ref.
ICP-MS	As, Pb, Cd, etc.	0.0003–2.47 mg·kg^−1^	peanuts	[30]
HPLC-ICP-MS	As	1.12 μg·kg^−1^	salted foods, sea salt	[31]
LA-ICP-MS	Hg	0.006 µg·g^−1^	mushrooms	[32]
	Se	0.3 µg·g^−1^		
GF-ETV-ICP-MS	Se	0.5 µg·kg^−1^	rice	[33]
	Cd	0.16 µg·kg^−1^		
FI-SPE-ICP-MS	Cd, Hg, Pb, etc.	0.8 ng·L^−1^–0.09 μg·L^−1^	rice	[34]
SPE-HPLC-AFS	Hg^2+^, MeHg, etc.	0.001–0.002 µg·L^−1^	water	[35]
ICP-AES	Hg	parts-per-trillion (ng·L^−1^)	cannabinoid-based products	[36]
ICP-MS-AES	Ag, As, Pb, etc.	below 3 mg·kg	turmeric	[37]
ICP-AES	Cd, As, Cu, etc.	0.008, 0.017, 0.0006, etc. (μg·L^−1^)	wheat and flour products	[38]
ICP-MS/AAS	Mn, Cr, As, etc.	0.1–23.2 mg·kg^−1^	honey	[39]
ICP-AES	Hg, As, Cd, etc.	1.80 × 10^−5^–2.17 × 10^−3^ mg·kg^−1^	milk powder	[40]
EcHG-AFS	Cd	0.05 ng·mL^−1^	drinking water	[41]
CVG-NDAFS	As	0.051 ng·mL^−1^	Chinese herbal foods	[42]
	Sb	0.034 ng·mL^−1^		
	Se	0.050 ng·mL^−1^		
	Hg	0.0058 ng·mL^−1^		
SS-ETV-AAS	Cd	70 pg·g^−1^	chocolate	[43]
TXRF	Mn, Ni, Rb, etc.	0.25–0.50 mg·kg^−1^	herbal infusion teas	[45]
HDXRF	As, Cd, Pb, etc.	0.072, 0.502, 0.063, etc. (mg·kg^−1^)	scallops	[46]
pXRF	Cr, Ni, As	20 mg·kg^−1^	fertilizers	[47]
pXRF-ICP-MS	Pb	2 mg·kg^−1^	spices	[48]
NIR-LIBS	Zn, Cu, Pb	4.3047, 4.9592, 8.3881 (mg·kg^−1^)	lilies	[50]

## 3. Spectroscopic Detection Methods Based on Fluorescent Probes

Although traditional methods can achieve accurate quantitative analysis of trace heavy metals in food, they often suffer from the disadvantages of complex operation processes, large amounts of reagents, long analysis times, expensive analytical instruments, and high requirements for professional and technical personnel [51]. In contrast, fluorescent probes can emit fluorescence at certain wavelengths when irradiated by ultraviolet light or visible light, with excellent photophysical properties such as high extinction coefficients, excellent quantum yields, and relatively long emission wavelengths. The characteristics of fluorescent probes can be changed with the environment so as to achieve effective detection of the measured substances with the advantages of good sensitivity, high selectivity, and short response times [52]. In recent years, fluorescent probe-based detection methods for heavy metals have been widely investigated by researchers. The focus of these methods has been the design and synthesis of specific probes with appropriate fluorophores and exploring the potential specific mechanisms. Herein, examples are summarized and classified, such as rhodamine, Schiff base, quinoline, coumarin, azoles, thiourea, tetraphenylene (TPE), thiophene, naphthoimide, etc.

### 3.1. Rhodamine-Based Fluorescent Probes

**Scheme 2:**
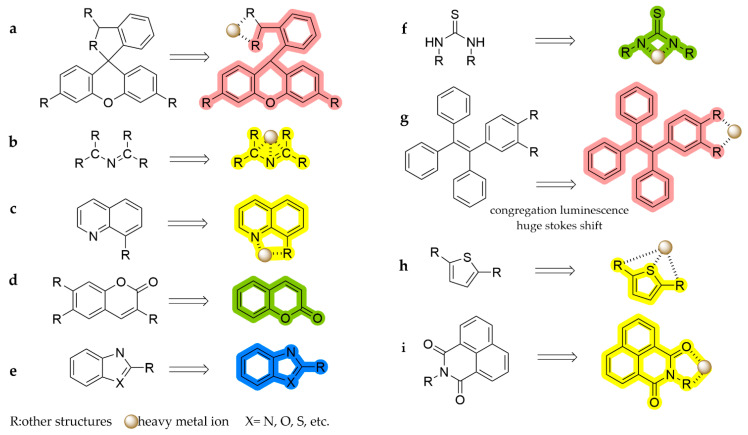
Recognition mechanisms of fluorophores: (**a**) rhodamine, (**b**) Schiff base, (**c**) quinoline, (**d**) coumarin, (**e**) azoles, (**f**) thiourea, (**g**) TPE, (**h**) thiophene, and (**i**) naphthoimide.

**Figure 1:**
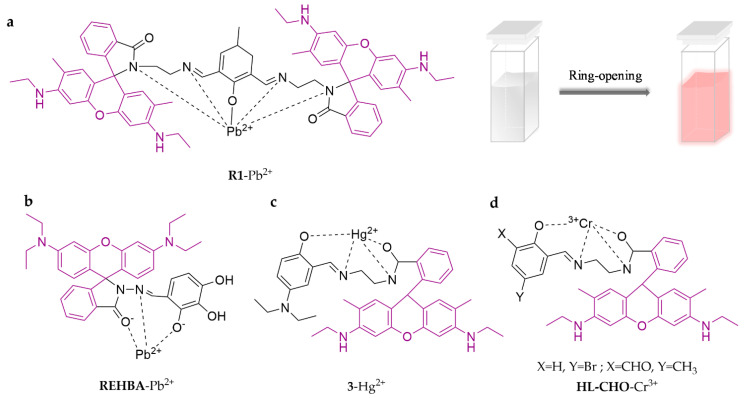
The structures of probes ((**a**–**d**) are **R1**, **REHBA**, **3** and **HL-CHO**, respectively).

Rhodamine spirolactam or spirolactone derivatives are non-fluorescent and colorless, while the ring opening of the corresponding spirolactam/lactone produces strong fluorescence emission and color change, which is an excellent characteristic for the preparation of fluorescence-enhanced probes (Scheme 2a). It is the most researched class of fluorescent probes by far (Figure 1). **R1** was designed as a selective probe for Pb^2+^ upon the combination of 2,6-diformyl-4-methylphenol with rhodamine 6G as the backbone [53]. The color of the working solution changes from light yellow to pink after recognition of Pb^2+^, which can achieve visual determination of Pb^2+^. The limit of detection is 2.7 × 10^−9^ M, and **R1** has been applied to detect lead in seafood, such as clams and scallops, with recoveries of 91.3–93.5%. Similarly, **REHBA** is a colorimetric fluorescent probe based on rhodaminohydrazine and 2,3,4-trihydroxybenzaldehyde that can effectively detect Pb^2+^ [54]. Its limit of detection is as low as 0.73 μM. It has been successfully applied in real water samples. A. Roy et al. [55] synthesized two novel rhodamine-based compounds. One (**3**) combined 5-diethylamino-2-hydroxy-benzaldehyde to detect Hg^2+^. The other (**HL-CHO**) combined 2,6-diformyl-4-methyl-phenol to detect Cr^3+^. Their detection limits are as low as 15.80 nM.

The current research on utilizing rhodamine-based probes for heavy metal detection in food primarily revolves around modifying various ligands on the spirolactam ring. This modification induces distinct fluorescence or color changes in the probes upon the addition of different metal ions, thereby facilitating the identification of heavy metals in food. Additionally, researchers have been focusing on developing portable detection devices that are practical and applicable in real-world scenarios, alongside the discovery of new probes.

#### 3.1.1. Fluorescent Probes to Detect Hg^2+^

**Figure 2:**
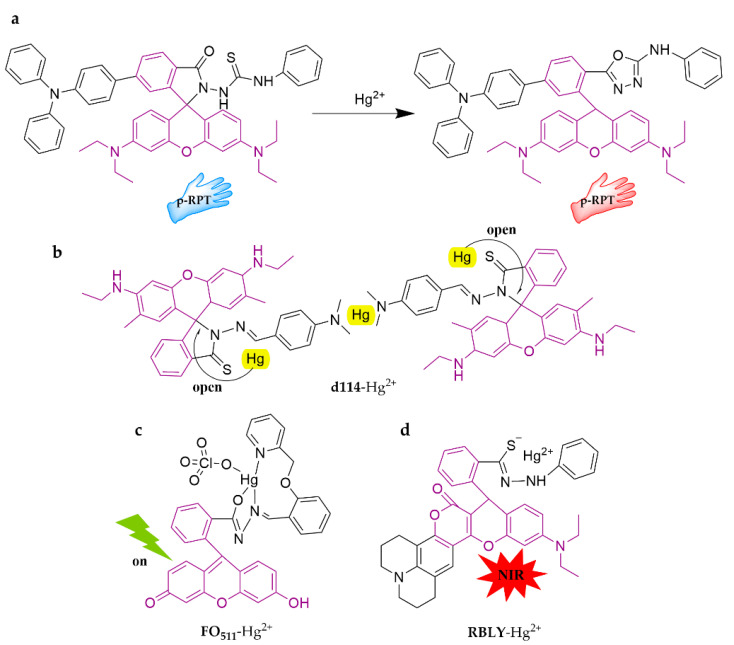
The structures of probes ((**a**–**d**) are **p-RPT, d114, FO_511_**, and **RBLY**, respectively).

As shown in Figure 2, a novel ratiometric fluorescent probe for detecting Hg^2+^, **p-RPT**, was designed with rhodamine derivatives and triphenylamine [56]. The probe could induce a visible color change (from blue to pink) when identifying Hg^2+^ in the THF/H_2_O (3:2, *v*/*v*) system and performed well in detecting mercury in water samples. Moreover, to accommodate field applications, researchers have developed hydrogel-coated paper sensors and flexible fluorescent gloves, respectively. The test paper is not only highly sensitive (the limit of detection is 1.2 × 10^−8^ M) but also can be stored for a long time. The flexible fluorescent gloves feature the same advantages, plus strong abrasion resistance. These two novel fluorescence-sensing tools were both successfully applied in the detection of Hg^2+^ in seafood, which provides a new perspective for a wearable sensing apparatus. **D114** and **FO_511_** are two other new rhodamine-lactam probes proposed for Hg^2+^ detection [57,58]. **D114** works well in the MeOH/H_2_O (1:1, *v*/*v*) system, with a limit of detection of 8.6 nM, and it could be contained with latex-coated slides for higher sensitivity (limit of detection as low as 0.5 nM). **FO511** is a fluorescein-2-(pyridin-2-ylmethoxy)-benzaldehyde conjugate. It can generate a 1:1 **FO511**-Hg^2+^ complex with a binding constant of (3.21 ± 0.05) × 10^4^ M^−1^ in HEPES (10 mM, pH = 7.2), which efficiently triggers Hg^2+^ with a limit of detection of 92.7 nM. As we all know, NIR fluorescent probes, emitting in the wavelength range of 650–900 nm, have the advantages of low energy, low background interference, high tissue penetration, and good imaging effect, and they have been a major hot topic of research in recent years. However, most rhodamine derivatives work in the 400–650 nm wavelength, restricting their application prospects. Therefore, **RBLY** was synthesized as a new NIR rhodamine-based probe to recognize Hg^2+^, which turns on strong fluorescence upon sensing Hg^2+^ in the EtOH/H_2_O (1:5, *v*/*v*) system, with a limit of detection of 0.34 μM [59]. It was successfully applied to water sample detection and live cell imaging.

#### 3.1.2. Fluorescent Probes to Detect Pb^2+^

**Figure 3:**
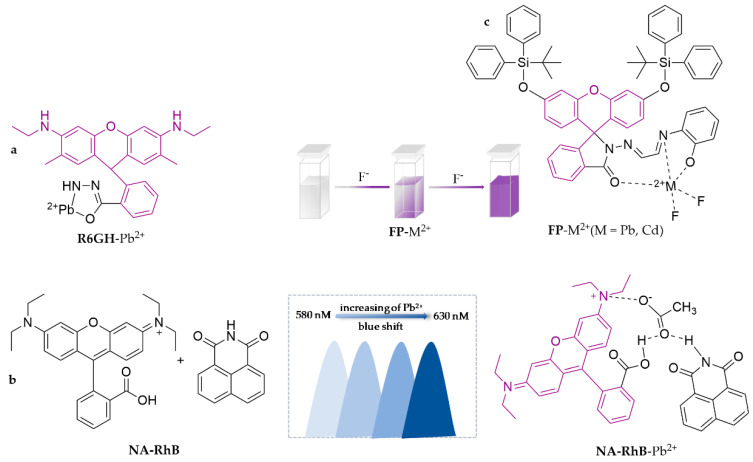
The structures of probes ((**a**) is **R6GH**, (**b**) is **NA-RhB**, (**c**) is **FP**).

For the efficient and convenient detection of Pb^2+^ in food, **R6GH** was synthesized and developed as an immobilized paper-based array sensor [60]. The sensor shows a good linear relationship response (R^2^ = 0.9851) in the concentration range of 0.05–6.0 μM, with a limit of detection of 0.02 μM. It has been successfully applied to aquatic detection with recoveries in the range of 84.0–102.0% and shows good linearity with the results of ICP-MS (R^2^ = 0.9915), which demonstrates it has strong practical applications. In recent years, fluorescence resonance energy transfer (FRET) techniques based on the principle of large emission wavelength shifts have shown broad applicability for improved heavy metal detection in food [61]. In particular, large emission wavelength shifts could avoid or minimize background interferences, resulting in high signal-to-noise ratios and high sensitivities with great potential. **NA-RhB** was presented as a novel sensor using the FRET principle, with 1,8-naphthalimide as the donor and rhodamine-B as the acceptor, and the peak wavelength was shifted to the shorter wavelength side (blue shift) with increasing concentrations of Pb^2+^ [62]. By observing the variations in emission peak wavelength shift (from 630.27 nm), a sensing probe for the detection of Pb^2+^ was developed, with a detection limit of 0.00001 g·L^−1^. **FP**, another a multifunctional colorimetric sensor based on rhodamine architecture, is capable of simultaneously sensing Pb^2+^ and Cd^2+^ with 2-(2-((2-hydroxyphenyl)imino)ethylidene) amino and tert-butyldiphenylsilyl modifications [63]. The color of the solution of **FP** in the EtOH/H_2_O (99:1, *v*/*v*) system turns light purple by visual observation after adding Pb^2+^ or Cd^2+^. Especially, when only F^−^ is present, the color of the **FP**-Pb^2+^ solution fades while that of the **FP**-Cd^2+^ solution darkens to purple, which displays the different spectral properties of the probe. The detection limits of **FP** are 0.42 μM for Pb^2+^ and 0.53 μM for Cd^2+^, and it has been successfully utilized for the determination of tap water with favorable linear recoveries. The specific structures of the probes are shown in Figure 3.

#### 3.1.3. Fluorescent Probes to Detect Cr^3+^

**Figure 4:**
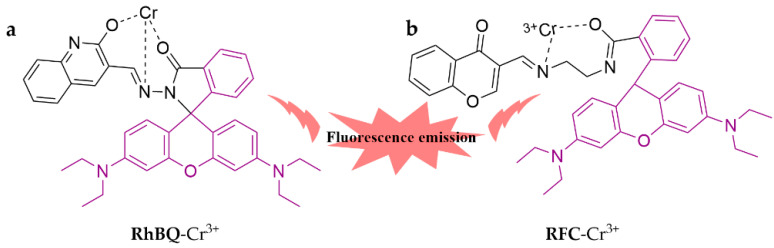
The structures of probes ((**a**) is **RhBQ** and (**b**) is **RFC**).

Figure 4 illustrates **RhBQ**, a chemosensor modified with 2-hydroxyquinoline-3-carbaldehyde, which exhibits both fluorescence emission and colorimetric sensitivity towards trivalent Cr^3+^ in ACN/H_2_O (9:1, *v*/*v*) medium [64]. The chemosensor has a detection limit of 2.12 × 10^−8^ M and has been successfully prepared as an assay test paper for convenient usage. A novel fluorescent probe, **RFC**, was synthesized by combining rhodamine and chromone through carbon–nitrogen conjugation for the purpose of detecting Cr^3+^ [65]. The fluorescent probe is effective in the MeOH/H_2_O (99:1, *v*/*v*) system and has a detection limit of 0.0052 ppm. Additionally, the probe exhibited remarkable anti-cancer activity against MCF-7 (breast cancer) cells with an IC_50_ value of 2.53 μM.

### 3.2. Schiff Base-Based Fluorescent Probes

**Figure 5:**
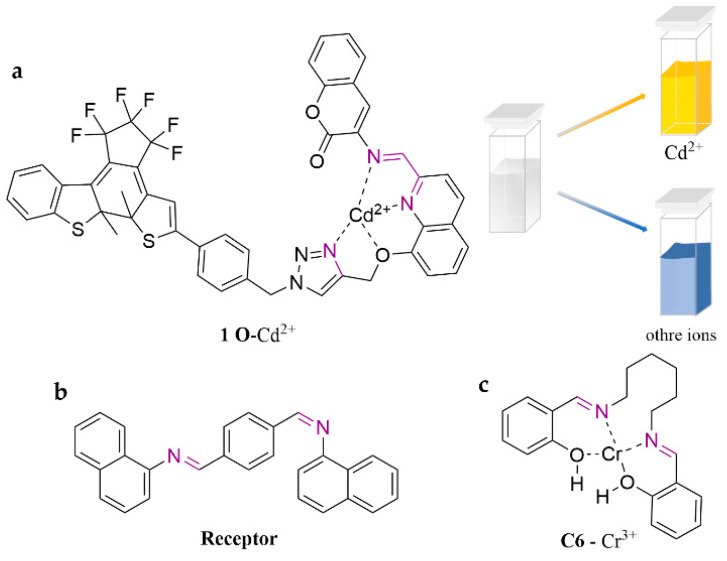
The structures of probes ((**a**) is **1 O**, (**b**) is **Receptor**, (**c**) is **C6**).

Schiff bases are organic compounds that contain imine or methylimine groups (C=N) with lone pairs of electrons in the hybrid orbitals of nitrogen atoms (Scheme 2b). These compounds are considered favorable ligands and exhibit photochromic characteristics. Therefore, there is significant interest in developing them as fluorescent probes for detecting heavy metals. As shown in Figure 5, a novel photochromic diarylethene containing a quinoline-linked 3-aminocoumarin Schiff base unit (**1 O**) was proposed [66]. The probe specifically responds to Cd^2+^ in the ACN system, which exhibits a visible color change (from dark cyan to golden yellow). **Receptor** is a Schiff base with structural modification of a naphthalene base, which is capable of detecting Cr^3+^ in aqueous solution, with a limit of detection of 3.92 μM [67]. It has performed successfully in cell imaging of zebrafish. Similarly, another probe, **C6**, was developed from 2,2′-(1E,1′E)-(hexane-1,6-diylbis-(azan-1-yl-1-ylidene))bis(methan-1-yl-1-ylidene)diphenol [68]. This probe exhibited excellent performance in tap water detection for Cr^3+^, with a limit detection of 13.3 µM.

Despite the development of numerous fluorescent probes based on the Schiff base framework, their practical applications are relatively rare due to their instability, weak specificity, and susceptibility to interference. However, in recent years, Schiff base structure-based fluorescent probes have overcome these limitations and have been successfully utilized for the detection of Cr^3+^, Cd^2+^, Pb^2+^, and so on.

#### 3.2.1. Fluorescent Probes to Detect Cr^3+^

**Figure 6:**
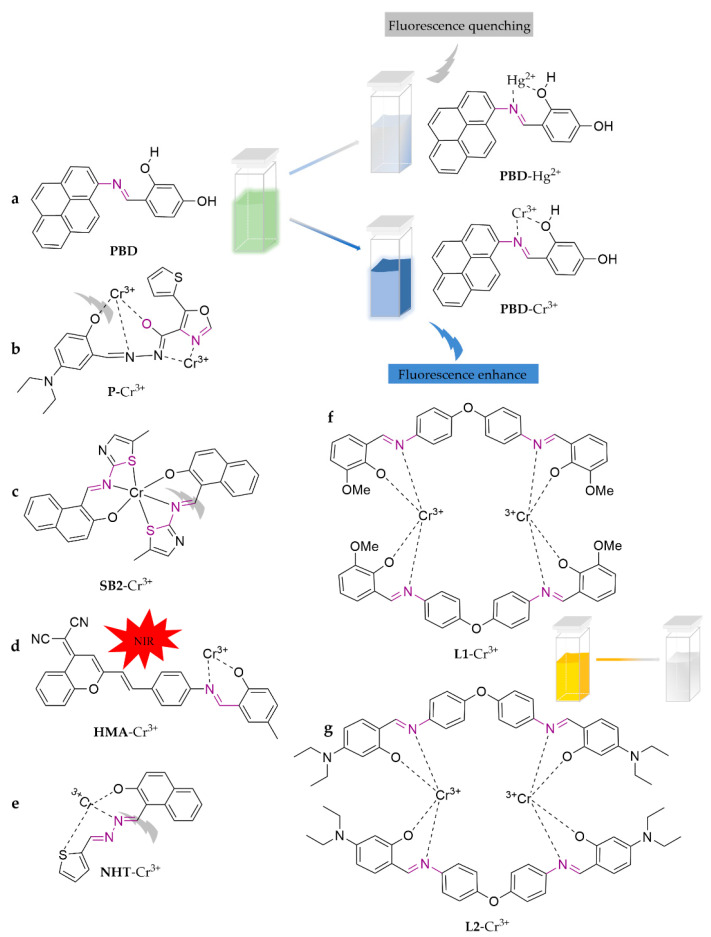
The structures of probes ((**a**–**g**)are **PBD**, **P**, **SB2**, **HMA**, **NHT**, **L1**, and **L2**, respectively).

As shown in Figure 6, **PBD** is a novel probe derived from pyrene, which exhibits strong fluorescence turn-on towards Cr^3+^ and fluorescence turn-off towards Hg^2+^ in the EtOH/H_2_O (1:1, *v*/*v*) system [69]. This unique sensitivity enables simultaneous detection of Cr^3+^ and Hg^2+^, with detection limits of 0.32 and 1.93 μM, respectively. The authors also tested the probe on tap water and soil, achieving favorable recoveries ranging from 96.0% to 105.7%. From 5-(thiophen-2-yl)oxazole-4-carbohydrazide and 4-diethylaminosalicylaldehyde, **P** was synthesized [70]. The sensor selectively quenches green fluorescence when determining Cr^3+^ in the DMF/H_2_O (9:1, *v*/*v*) system, with a limit of detection of 9.82 × 10^−9^ M. This sensor has been successfully utilized for monitoring targeted ions in actual water samples. A novel thiazole-based fluorescent and colorimetric Schiff base chemosensor, **SB2**, was designed to achieve highly sensitive, selective, and efficient identification [71]. It operates in the MeOH/H_2_O (3:1, *v*/*v*) system and exhibits fluorescence turn-on and a colorimetric response (from yellow to colorless), with detection limits as low as 0.5 µM. In addition, **SB2** has been successfully applied in soil samples with recoveries ranging from 95.00 ± 0.50% to 99.00 ± 0.14%. The dicyanomethylene-4H-pyran (DCM) unit is one of the most commonly used NIR fluorophores, with a large Stokes shift and high light stability [72]. Therefore, **HMA** was designed containing DCM [73]. The process of determining Cr^3+^ along with the NIR fluorescence enhancement in the DMSO/H_2_O (9:1, *v*/*v*) system yielded a detection limit of 5.63 × 10^−7^ M. This probe has been successfully applied to the detection of water samples. A thiophene-substituted naphthyl hydrazone derivative, **NHT**, was synthesized using a one-step route for the detection of trivalent Cr^3+^ [74]. It shuts off fluorescence in HEPES buffer (0.2 M, pH = 7.2), with a limit of detection of 41 nM. The modification enhances probe solubility and biocompatibility, which allowed its further application in the bioimaging of PC3 cells (human prostate cancer cells). R. Chandra et al. [75] designed and synthesized two salicylaldimine-functionalized dipodal bis Schiff base fluorescent colorimetric chemosensors, **L1** (4-(2-hydroxy-3-methoxybenzyl-ideneamino)phenyl) and **L2** (4-phenylimino)4-diethylsalicyl-al-dehyde). They were able to distinguish Cr^3+^ in the ACN/H_2_O (1:1, *v*/*v*) system. **L1** shows a binding constant of 1.26 × 10^5^ M^−1^ with a detection limit of 1.12 × 10^−7^ M, while **L2** shows a binding constant of 3.0 × 10^5^ M^−1^ with a detection limit of 7.73 × 10^−7^ M. Researchers also successfully developed a visible colorimetric kit and applied it to rapid detection in real water samples.

#### 3.2.2. Fluorescent Probes to Detect Cd^3+^

**Figure 7:**
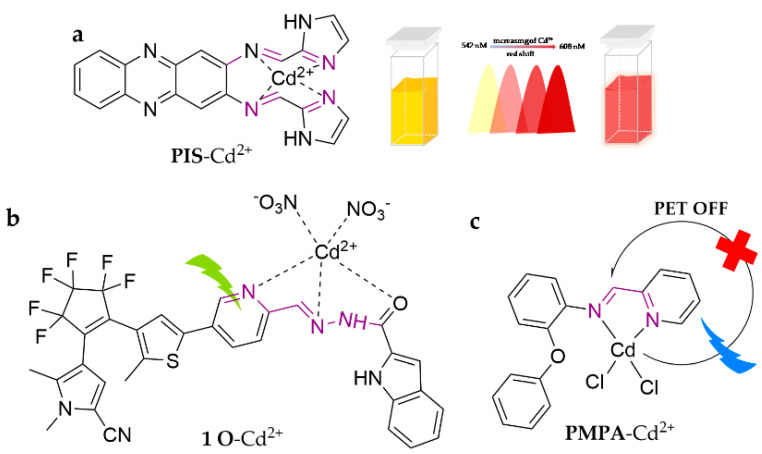
The structures of probes ((**a**) is **PIS**, (**b**) is **1 O**, (**c**) is **PMPA**).

In Figure 7, **PIS** was developed as a novel ratiometric phenazine-imidazole-Schiff base fluorescent probe [76]. The probe works in the ACN/HEPES buffer (10 mM, pH = 7.4) (1:4, *v*/*v*) system. It evokes a significant fluorescence color change (from yellow to orange-red), followed by a large red shift of the Stokes peak (542 to 608 nm). Through monitoring the variation in the fluorescence ratio at 608 and 542 nm with increasing Cd^2+^ concentration at Ex.406 nm, this probe can detect Cd^2+^ with detection limits as low as 2.10 × 10^−8^ M. In addition, this probe has been successfully applied to cell imaging of zebrafish larvae and Chinese rice locust cells with specific potential. Z. Wang et al. [77] reported a different Schiff base fluorescent probe modified with diaryl ethylene derivatives (**1 O**). The probe detects Cd^2+^ in THF medium, evoking photochromism (fluorescence color changed from black to green) and a fluorescence turn-on effect, accompanied by a significant red shift of the fluorescence emission peak (up to 105 nm). The sensor has a binding constant of 4.36 × 10^4^ M^−1^ and a detection limit of 5.74 × 10^−7^ M. It has been successfully utilized for accurate detection in tap water samples. **PMPA** was designed and synthesized as a probe customized by pyridine derivatives with the photoinduced electron transfer (PET) mechanism and chelation-enhanced fluorescence (CHEF) properties for the selective detection of Cd^2+^ in ACN [78]. The probe exhibits a wide pH range flexibility (5–9), quick response time (3 min), excellent sensitivity (LOD = 0.12 mM), and favorable reversibility. Furthermore, it has been successfully applied to determine real water samples.

#### 3.2.3. Fluorescent Probes to Detect Pb^2+^

**Figure 8:**
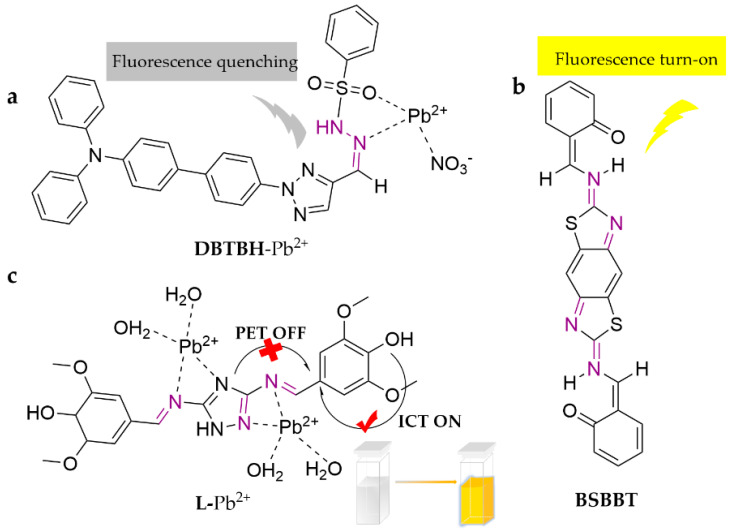
The structures of probes ((**a**) is **DBTBH**, (**b**) is **BSBBT**, (**c**) is **L**).

**DBTBH** is a Schiff base fluorescent probe amended with a benzenesulfonylhydrazone derivative [79]. The probe exhibits rapid, efficient, and sensitive response to Pb^2+^ in THF/Tris-HCl buffer (10 mM, 1 mM KI, pH = 7.4) (1:9, *v*/*v*), with a detection limit of 4.49 × 10^−8^ M. The recognition process exhibits excellent optical properties of aggregation-induced emission enhancement (AIEE) and intramolecular charge transfer (ICT). It has been implemented in real water samples. Another novel symmetric tridentate probe, **BSBBT**, facilely equipped with a benzodiazole and salicylaldehyde structure, is able to detect Pb^2+^ in the DMSO/H_2_O (3:7, *v*/*v*) system with a detection limit of 2.23 × 10^−6^ M [80]. The photophysical analysis showed an enhanced emission in the aggregates with excellent AIEE properties in solution or solid state. The tautomerization of keto/enol or hydrogen bonding interactions in the molecule are the origin of the large Stokes shifts and d excited-state intramolecular proton transfer (ESIPT) properties, which enable it to be a useful material for photonic devices. **L**, a Schiff base modified with triazole derivatives, was proposed as a new colorimetric chemosensor [81]. The imine-attached triazole moiety contains multiple pockets suitable for metal coordination, whereas the phenol containing two methoxy groups acts as a signaling sub-unit, which enhances the ICT and PET processes. It recognizes Pb^2+^ in the MeOH/Tris buffer (1:1, *v*/*v*) system, with sensible colorimetric sensing capability (from colorless to light yellow), strong fluorescence turn-on, and a detection limit of 9 × 10^−7^ M. This sensor has been successfully employed in real water samples. The specific structures of the probes are shown in Figure 8.

### 3.3. Quinoline-Based Fluorescent Probes

Quinoline and its derivatives are common metal ion chelators, with rigid structures, large conjugation complexes, and excellent performance in aqueous solvents. Normally, the fluorescence is enhanced after complexing with metal ions, so quinolines have often been employed in the development of fluorescent probes in recent years (Scheme 2c).

#### 3.3.1. Fluorescent Probes to Detect Cd^3+^

**Figure 9:**
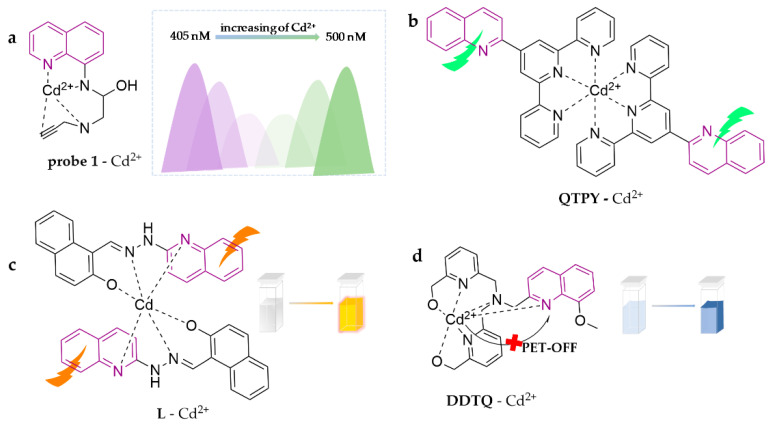
The structures of probes ((**a**–**d**)are **probe 1**, **QTPY**, **L** and **DDTQ**, respectively).

As shown in Figure 9, **probe 1** was synthesized as a novel ratiometric fluorescent probe via combining the fluorescent group of 8-aminoquinoline with an amide group on the chelating site of propargylamine [82]. The probe can detect Cd^2+^ in ACN through observation of the variation in the fluorescence ratio response between 500 and 405 nm under Ex.335 nm with increasing Cd^2+^ concentration and has a detection limit of 0.055 μM. In addition, the researchers explored **probe 1** as test strips, which have been successfully applied in water samples, bean sprouts, etc. **QTPY** was reported as a ratiometric fluorescent probe, 4′-quinolin-2-yl-[2, 2′; 6′, 2′′] terpyridine [83]. The probe is capable of detecting Cd^2+^ in the DMF/H_2_O (4:6, *v*/*v*) system based on the intramolecular electron transfer mechanism. The fluorescence intensity increases significantly with increasing Cd^2+^ concentration at 520 nm and shows a favorable linear relationship with a detection limit of 3.5 × 10^−8^ M. By combining 2-hydroxy-1-naphthaldehyde and 2-hydrazinoquinoline, **L** was developed [84]. The free **L** shows no fluorescence due to the companied PET and C-N isomerization mechanism, while when it is coordinated with Cd^2+^ in the ACN/H_2_O (8:2, *v*/*v*) system, free ligand C-N isomerization is restricted and the CHEF phenomenon induces enhanced fluorescence. It also evokes a variation in color from colorless to crimson yellow. The probe forms a 2:1 complex with Cd^2+^ with a binding constant of 1.77 × 10^5^ M^−1^ and a detection limit of 14.8 nM. It performed well in detection of different water samples. A newly designed and synthesized quinoline-based chemosensor, **DDTQ**, was reported for the detection of Cd^2+^ in aqueous media [85]. It shows excellent luminescence behavior following the PET and CHEF mechanisms, which yields significantly enhanced fluorescence at 445 nm, with a detection limit of 126 nM. Furthermore, the probe performs with low toxicity and excellent biocompatibility, which motivated the researchers to explore the biofluorescence imaging applications in living cells, as well as illustrating the potential for its real-life applications.

#### 3.3.2. Fluorescent Probes to Detect Pb^2+^ and Hg^2+^

**Figure 10:**
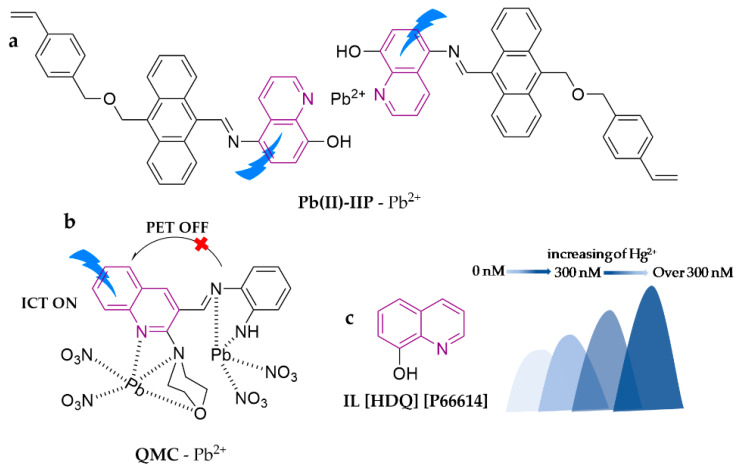
The structures of probes ((**a**) is **Pb(II)-IIP**, (**b**) is **QMC**, (**c**) is **IL [HDQ] [P66614]**).

A novel fluorescent probe, **Pb(II)-IIP**, was created by modifying a 5-amino-8-hydroxyquinoline-type probe with styryl anthracene derivatives. The probe was then manufactured into microbeads through polymerization with ethylene glycol dimethacrylate (EGDMA) [86]. The highly mesoporous material is capable of detecting Pb^2+^ in pure water, hence turning on the fluorescence with a detection limit of 2.1 μg∙L^−1^. The method has been successfully applied in tap water, mineral water, and seawater sample determination. Another novel quinoline-morpholine conjugate, **QMC**, was also developed [87]. The probe displays highly selective detection of Pb^2+^ in the ACN/H_2_O (1:1, *v*/*v*) system, exhibiting a large blue shift and fluorescence enhancement during the assay. It is the first fluorescent chemosensor to employ blue shift tuning via the ICT process for the detection of Pb^2+^. Furthermore, **QMC** exhibited no interference among alkali metal, alkaline earth metal, and transition metal ions, with a detection limit of 13 μM. The researchers have also extended the usage of the probe for the detection of Pb^2+^ in milk and red wine, with a recovery between 93.7%–103.2%. S. Che et al. [88] presented a novel ionic fluorescent probe, **IL [HDQ] [P66614]**, consisting of a functional quinoline-based compound. It works in ethanol solution for Hg^2+^ detection. Interestingly, the probe exhibits two different fluorescence signals in the presence of various concentrations of Hg^2+^. The fluorescence intensity first gradually increases when the Hg^2+^ concentration is increased from 1 to 300 nM, accompanied by a blue shift of the emission peak from 413 to 388 nm, while the fluorescence intensity gradually decreases with the peak continuing to blue shift from 300 to 103 nm when the Hg^2+^ concentration continues to accumulate. The researchers hypothesized and confirmed this was due to two interaction modes: electrostatic attraction and chemical conjugation. First, the probe mainly engages in electrostatic attraction to form a large complex regime in the presence of a tiny amount of Hg^2+^ while the phototransfer effect of the functional group (the [HDQ] [P66614] part) is hindered. Then, the probe engages in chemical conjugation with additional Hg^2+^, the original complex regime is disrupted, and new quaternary anion complexes appear. Therefore, the probe is useful as a fluorescent colorimetric probe for real-time monitoring of Hg^2+^ within a concentration range of 1–10^3^ nM and the detection limit is 0.8 nM. It has been successfully employed in real water samples, fish, and shrimp. Test strips based on **IL [HDQ] [P66614]** have been developed to further enrich its application prospects in various industries. The specific structures of the probes are shown in Figure 10.

### 3.4. Coumarin-Based Fluorescent Probes

The coumarin parent is not fluorescent, while the derivatives of coumarin show intense fluorescence, containing a push–pull electron system formed through the incorporation of different electron-absorbing and electron-donating groups. The derivatives possess the advantages of structural adjustment, high quantum yield, large Stokes shift, and excellent photostability, which can sequester metal ions to enhance the fluorescence performance. Therefore, they can be widely used to compose metal ion fluorescent probes for the detection of heavy metal ions (Scheme 2d).

#### 3.4.1. Fluorescent Probes to Detect Hg^2+^

**Figure 11:**
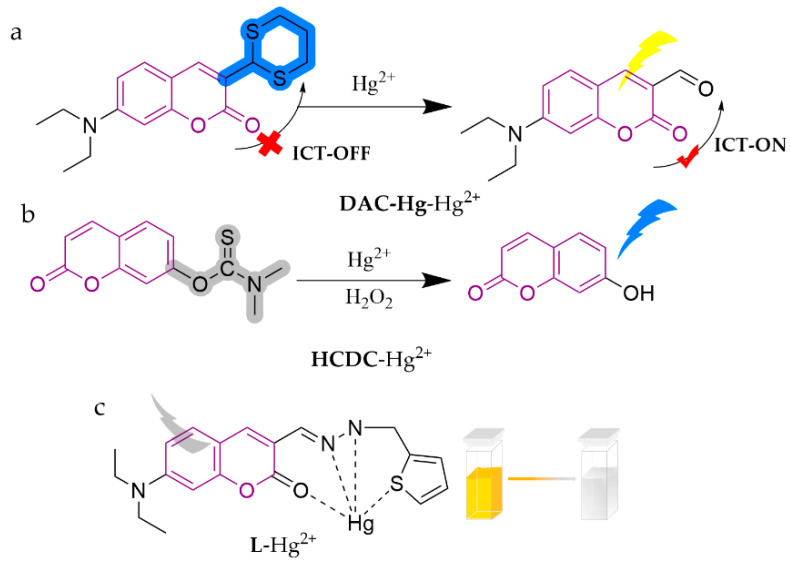
The structures of probes ((**a**) is **DAC-Hg**, (**b**) is **HCDC**, (**c**) is **L**).

As shown in Figure 11, **DAC-Hg** is a coumarin-based fluorescent probe modified with sulfhydryl groups [89]. It can identify Hg^2+^ with high selectivity and sensitivity in PBS buffer, displaying a “turn-off” fluorescence effect. The color varies from cyan to nearly weak yellow (non-fluorescent), with a detection limit of 5.0 nM. The application of this method in analyzing environmental and seafood samples yielded satisfactory results (range of 96.2–105.0%), offering a promising approach for Hg^2+^ detection and potential applications in sensing other heavy and transition metal ions. **HCDC** was amended with dimethylthiocarbamate moieties, serving as a sensor for detecting Hg^2+^ in HEPES buffer (5 M, pH = 7.4) medium [90]. This sensor triggers a switch from the thioester group to the ester group of the probe and turns on blue fluorescence. Notably, the response changes become significantly more pronounced under the aid of H_2_O_2_ (about 350-fold stronger), with a detection limit of 0.3 nM. The researchers further accomplished real water sample determination. J. Isaad et al. [91] constructed a novel coumarin hydrazone fluorescent probe, **L**, from 7-diethylamino coumarin-3-carbaldehyde and 2-thiophenethylhydrazine. It is capable of detecting Hg^2+^ in bis-tris buffer (10 mM, pH = 7.0, 0.5% DMSO), rendering a red shift in the emission wavelength from 423 to 508 nm. The burst of fluorescence intensity at 516 nm, accompanied by a distinct color change from yellow to red, is clearly visible to the naked eye and is related to ICT and d-d transitions of the metal cations. The detection limit is 5.15 nM and it has been successfully applied to water samples.

#### 3.4.2. Fluorescent Probes to Detect Cd^2+^ and Pb^2+^

**Figure 12:**
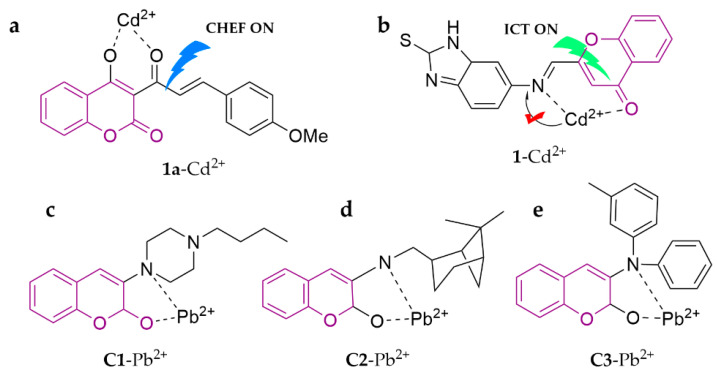
The structures of probes ((**a**–**e**) are **1a**, **1**, and **C1–C3**, respectively).

A novel chemochromic sensor composed of chalcone-modified coumarin, **1a**, was proposed for selective and sensitive recognition toward Cd^2+^ in HEPES buffer medium (20 mM, ACN/H_2_O, 3:7, *v*/*v*, pH = 7.0) [92]. Based on the CHEF mechanism, this probe performs a significant color change (from yellow to colorless) along with a notable fluorescence turn-on, with a detection limit of 5.84 × 10^−8^ M. The novel coumarin-type Schiff base sensor, known as probe **1**, can differentiate Cd^2+^ in the THF/H_2_O (1:1, *v*/*v*) system with a low detection limit of 0.114 μM, which is attributed to the inhibition of the C=N isomerization effect [93]. Aminocoumarins exhibit an interesting behavior in polar solvents, displaying intramolecular charge transfer (ICT) and leading to charge separation in the excited state due to their push–pull character as a π-conjugated system. This ICT results in a red shift in the absorption of the receptor bands and a decrease in photon emission efficiency, confirming the binding of metals [94]. Keeping all these advantages of aminocoumarins in view, in continuation with work in this ongoing vibrant field, three amino-substituted coumarin receptors (**C1–C3**) were reported that show selective and sensitive binding with Pb^2+^ in ACN [95]. In addition, all of the substituted amine derivatives were common bioactive substances, which facilitates the further application of the probes for human metabolomics studies. The specific structures of the probes are shown in Figure 12.

### 3.5. Fluorescent Probes Based on Imidazole, Benzoxazole, Pyrazole, and Other Azoles

Azoles are pentacyclic compounds containing nitrogen. The hybrid atoms in pentacyclic compounds contain lone pairs, leading to the pentacyclic compounds easily forming hydrogen and coordination bonds and having strong electron affinity potential. There are many electroluminescent devices developed based on them, which can emit high-energy blue light or purple light. Thus, azoles are important photosensitive substances. Recently, numerous fluorescent probes have been developed based on imidazole, benzoxazole, pyrazole, and other components for the detection of heavy metals (Scheme 2e).

#### 3.5.1. Imidazole-Based Fluorescent Probes

Imidazole is a collective term for pentameric aromatic heterocyclic compounds containing two interposition nitrogen atoms in the molecular structure, owing to the participation of the undisposed electron pair of the nitrogen atom in the ring in cyclic conjugation. These characteristics induce lower electron density of the nitrogen atom, easier departure of the hydrogen on this nitrogen atom, and more effective binding to metal ions.

**Figure 13:**
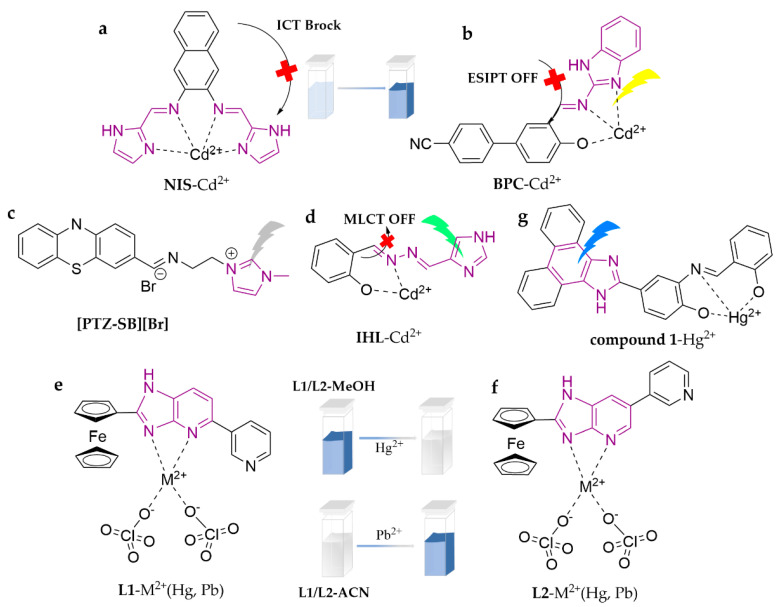
The structures of probes ((**a**–**g**) are **NIS**, **BPC**, **[PTZ-SB][Br]**, **IHL**, **L1**, **L2**, and **compound 1**, respectively).

As shown in Figure 13, probe **NIS** was synthesized by one-step reaction between 2,3-naphthalenediamine and imidazole-2-carboxaldehyde [96]. It selectively and sensitively recognizes Cd^2+^ in HEPES buffer (EtOH/H_2_O = 9:1, *v*/*v*, pH = 7.4), entraining the charge transfer and chelation effects to turn on the fluorescence internally, yielding a blue shift of the maximum emission peak of 74 nm, presumably due to the combined effects of the ICT and CHEF mechanisms. By monitoring the variation in the fluorescence intensity ratio at 398 and 472 nm (Ex.342 nm), **NIS** is able to monitor Cd^2+^ with a detection limit of 3.87 × 10^−7^ M. It has also been successfully utilized for the detection of human liver cancer cells (SMMC-7721), zebrafish, and other in vivo tissues. **BPC** is an imidazole-based fluorescent probe modified with 4-cyanobiphenyl [97]. It can recognize Cd^2+^ in ACN/Tris–HCl buffer (3:2, *v*/*v*, pH = 7.4), eliciting a significant color change (from colorless to yellow), fluorescence turn-on at 547 nm, and has a detection limit of 1.05 × 10^−8^ M. The 1:1 complex formation between **BPC** and Cd^2+^ can block both isomerization of C-N and the ESIPT process; meanwhile, it enhances the molecular rigidity, resulting in chelation-enhanced fluorescence (CHEF). A highly green fluorescent phenothiazine-based imidazolium ionic liquid sensor, **[PTZ-SB][Br]**, was synthesized [98]. The sensor can recognize Cd^2+^ in the THF/H_2_O (1:9, *v*/*v*) system, achieving an incredible 38.1-fold increase in fluorescence intensity with a detection limit of 3.8 × 10^−7^ M. Investigators demonstrated that this change was due to the aggregation-induced enhanced emission (AIEE) phenomenon. In order to broaden the applicability of the sensor in field testing, a fluorescent paper strip has also been developed and successfully applied to water sample detection. **IHL** is a new salicylhydrazone-anchored imidazolyl derivative [99]. This probe exhibits excellent biological potential for the identification of Cd^2+^ in the DMSO/H_2_O (9:1, *v*/*v*) system, showing a new absorbance at the 354 nm wavelength based on metal-to-ligand charge transfer (MLCT) and C=N isomerization. The detection limit is 0.4 × 10^−10^ M and it has been successfully employed in conducting in vitro fluorescence imaging of zebrafish embryos. **L1** and **L2** are two probes modified with ferrocene and pyridine, which can switch the selective recognition from Hg^2+^ to Pb^2+^ via switching the solvent system [100]. **L1** and **L2** display highly selective recognition of Hg^2+^ over a red shift in the MeOH/H_2_O (1:1, *v*/*v*) system, with immense brightness and remarkable fluorescence quenching, and detection limits of 7.6 × 10^−6^ and 6.7 × 10^−6^ M, respectively. Meanwhile, a similar UV response was induced by adding Pb^2+^ in the ACN system, but revealing great fluorescence enhancement (CHEF = 25 for **L1** and 33 for **L2**) and detection limits of 8.5 × 10^−6^ and 2.5 × 10^−6^ M, respectively. The PET mechanism is responsible for their fluorescence changes. **Compound 1** was investigated based on 4-hydroxy-3-nitrobenzaldehyde and 9,10-phenanthrenequinone [101]. It possesses a highly sensitive (detection limit of 45.76 nM) and rapid response (within 5 s) towards Hg^2+^ in the DMF/PBS buffered solution (1:4, *v*/*v*, pH = 7.4) system, accompanied with variation in the turn-on blue fluorescence. In addition, researchers have successfully employed it in the determination of real water samples, with recoveries of 91–106% and RSDs of less than 2.5%.

#### 3.5.2. Benzoxazole-Based Fluorescent Probes

**Figure 14:**
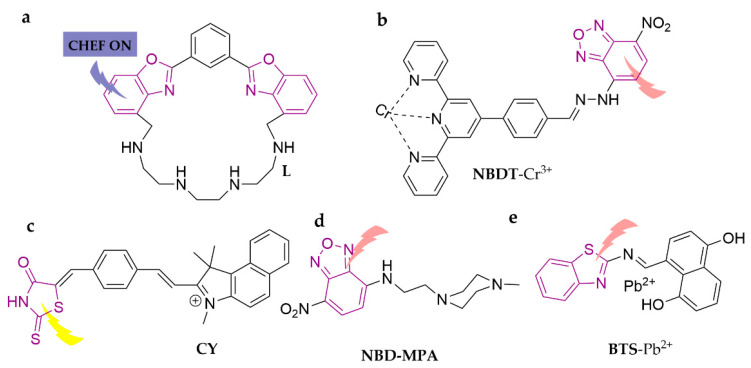
The structures of probes ((**a**–**e**)are **L**, **NBDT**, **CY**, **NBD-MPA**, and **BTS**, respectively).

Benzoxazole is similar to imidazole in its structural characteristics, with excellent metal ion-chelating properties that offer broader application prospects. **L** is a cyclophane macrocycle containing the 1,3-bis(benzo[d]oxazol-2-yl)phenyl(BBzB) fluorophore and an aliphatic tetra-amine chain to form the macrocyclic skeleton [102]. The sensor is a selective PET-regulated chemosensor for Cd^2+^ in the ACN/H_2_O (4:1, *v*/*v*) system, with detection limits as low as 0.03 ppm. An NBD-based (4-chloro-7-nitrobenzo-2-oxa-1,3-diazole) fluorescent probe, **NBDT**, was fabricated with extraordinary specificity and sensitivity for Cr^3+^ [103]. It works in the DMSO/H_2_O (9:2, *v*/*v*) system over a wide pH range and cycle stability, showing a color change (from purple to red) and a blue shift (from 548 nm to 522 nm). The detection limit is 0.041 μM. In addition, researchers have successfully applied **NBDT** in detecting exogenous Cr^3+^ in MDA-MB-231 (human breast cancer cells), HepG2 (human liver cancer) cells, and zebrafish embryonic cells. **CY** is an imidazole class fluorescent probe structured with a semi-flowering anthocyanine [104]. It specifically identifies Hg^2+^ in the DMSO/H_2_O (7:3, *v*/*v*) system, displaying a fluorescence-on response with a detection limit of 1.61 × 10^−7^ M. This probe was successfully employed in real water sample detection. A mercury ion-specific fluorescent probe, **NBD-MPA**, was developed by a simple one-step reaction of commercial substrates of 4-chloro-7-nitro-2,1,3-benzoxadiazole and 1-(2-aminoethyl)-4-methylpiperazine [105]. The probe senses Hg^2+^ in ACN/HEPES buffer solution (1:9, *v*/*v*), provoking a red shift of the weak emission peak at 550 to 580 nm and turning on strong fluorescence, while the color shifts from yellow to pink independent of other metal cations, with a detection limit of 9.2 × 10^−7^ M. Researchers further employed the probes successfully for Hg^2+^ quantification in tap water, soil, green tea, and sea shrimp, showing promising applications. **BTS**, a probe based on 4-(benzo [d] thiazol-2-yldiazenyl) naphthalene-1,5-diol, was synthesized [106]. The sensor can recognize Pb^2+^ in the DMSO/H_2_O (1:4, *v*/*v*) system, displays a marked spontaneous color change from blue to pink, and has a detection limit of 0.67 µM. In addition, test strips have also been developed for its utilization as a rapid colorimetric chemical sensor, which have been successfully applied in real water samples. The specific structures of the probes are shown in Figure 14.

#### 3.5.3. Pyrazole-Based Fluorescent Probes

**Figure 15:**
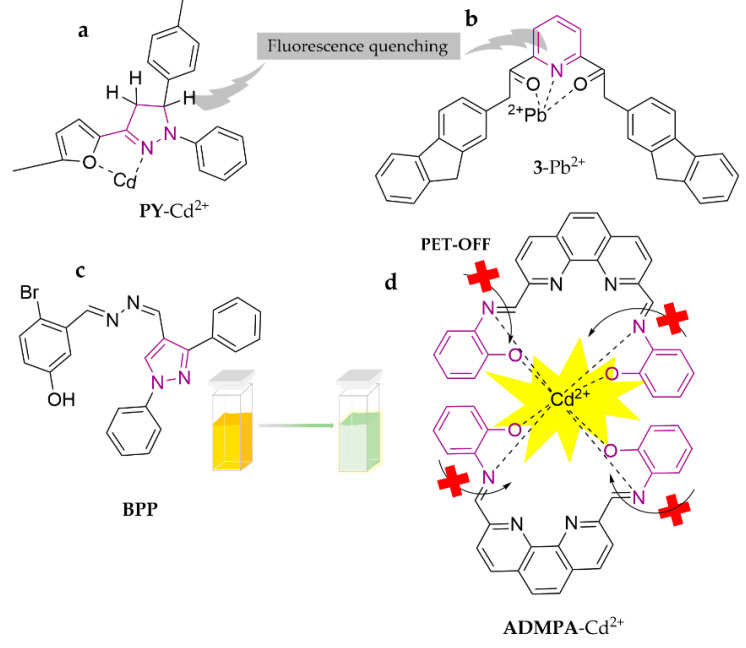
The structures of probes ((**a**–**d**) are **PY**, **3**, **BPP** and **ADMPA**, respectively).

Pyrazoles and their derivatives are considered as promising fluorescent compounds due to their excellent photophysical properties, including high fluorescence quantum yields and blue light emission with long fluorescence lifetimes. As shown in Figure 15, a fluorescent sensor based on pyrazoline-based compound 5-(4-methylphenyl)-3-(5-methylfuran-2-yl)-1-phenyl-4, 5-dihydro-1H-pyrazole, **PY**, has been synthesized for the selective detection of Cd^2+^ in water samples [107]. The sensor analyzes Cd^2+^ in MeOH/PBS buffer (pH = 7) system, induces fluorescence quenching with a detection limit of 0.09 μM, and has been applied successfully for the determination of water samples (tap water, river water and bottled water). The dominant process for fluorescence quenching is attributed to the intermolecular PET mechanism. A new tripodal fluorogenic and chromogenic receptor, 5-bromosalicyl hydrazone appended pyrazole, **BPP**, has been designed and synthesized [108]. The fluorescent probe can recognize Cd^2+^ in the DMSO/H_2_O (9:1, *v*/*v*) system, with the color of the solvent switching from light yellow to light yellow-green, and a detection limit of 0.02 nM. In addition, the outcome of fluorescence imaging assays in HeLa cells and zebrafish embryos suggested that **BPP** could be useful in biological systems as a potential chemical device. **ADMPA** was proposed as another novel highly sensitive and rapid-response fluorescent probe based on o-aminophenol and 2,9-dimethyl-1,10-o-phenanthroline [109]. The probe can recognize Cd^2+^ in the DMF/H_2_O (3:7, *v*/*v*) system and induce probe fluorescence to turn on, owing to the suppression of PET and CHEN isomerization. The detection limit is as low as 29.3 nM. This method has been successfully utilized for the determination of real water samples with satisfactory recoveries. H. Rahimi et al. [110] demonstrated a novel fluorescent probe, **3**, containing pyridine-2, 6-dicarboxamide. The probe recognizes Pb^2+^ in ACN and shows fluorescence quenching, with a detection limit of 2.31 × 10^−6^ M. Ease of synthesis and impressive sensitivity and selectivity are the main features of this cation chemosensor.

### 3.6. Thiourea-Based Fluorescent Probes

**Figure 16:**
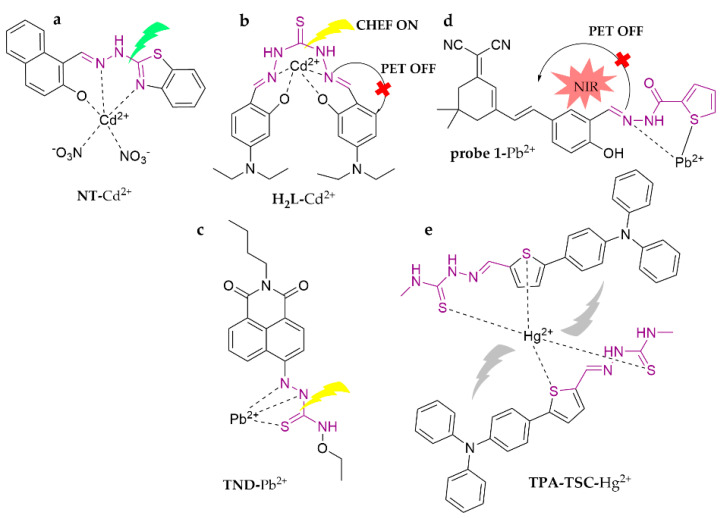
The structures of probes ((**a**–**e**) are **NT**, **H_2_L**, **TND**, **probe 1** and **TPA-TSC**, respectively).

The thiourea structure contains thiocarbonyl C=S, which shows differences in shape and energy between the 2p orbital of the C-atom and the 3p orbital of the S-atom in thiocarbonyl, with less orbital overlap. It allows a weaker Π-bond in the thiocarbonyl group and unique coordination ability (Scheme 2f). As shown in Figure 16, a novel fluorometric probe, **NT**, was designed to detect Cd^2+^ in ACN [111]. It could inhibit immediate strong green fluorescence and turned on intensive green fluorescence owing to the suppression of C=N isomerization by Cd^2+^. S. S. Samanta et al. [112] presented a fluorescent probe, **H_2_L**, which was capable of recognizing Cd^2+^ in MeOH, turning on strong fluorescence with a detection limit of 2.67 × 10^−8^ M, and was applicable in food, beverage, and environmental detection. A novel naphthalimide derivative, **TND**, modified with aminothiourea, was presented for acquiring Pb^2+^ in the ACN/H_2_O (1:1, *v*/*v*) system [113]. It displays a clear color change (from light green to yellow) and turn-on fluorescence based on the superiorities of naphthalimide, which has the properties of great stability and easy modification. The detection limit is 4.7 nM and it has been successfully employed in the detection of water samples with a spiked recovery of 100.54–113.68%. An NIR fluorescent probe for the fast determination Pb^2+^, **probe 1**, was synthesized based on a carbonyl hydrazide derivative modified with a dicyanoisophorone backbone [114]. The probe with the thiophene-2-carbohydrazide group is capable of capturing Pb^2+^ in the ACN/EtOH/HEPES system (1:1:2, *v*/*v*/v, pH = 7.0), with a maximum emission wavelength of 670 nm. It has been successfully utilized in real water samples, with a detection limit of 1.65 nM. A novel triamine-thiophene-aminothiourea fluorescent probe, **TPA-TSC**, was proposed for excellent selectivity toward Hg^2+^ in the ACN/PBS system (1:1, *v*/*v*, pH = 7.4), with the lowest detection limit (0.14 nM) yet reported [115]. It exhibited a turn-off response by forming a stable complex for a brief period (<30 s). In addition, the investigators successfully applied the probes to perch, sailfish, and different water samples.

### 3.7. TPE-Based Fluorescent Probes

**Figure 17:**
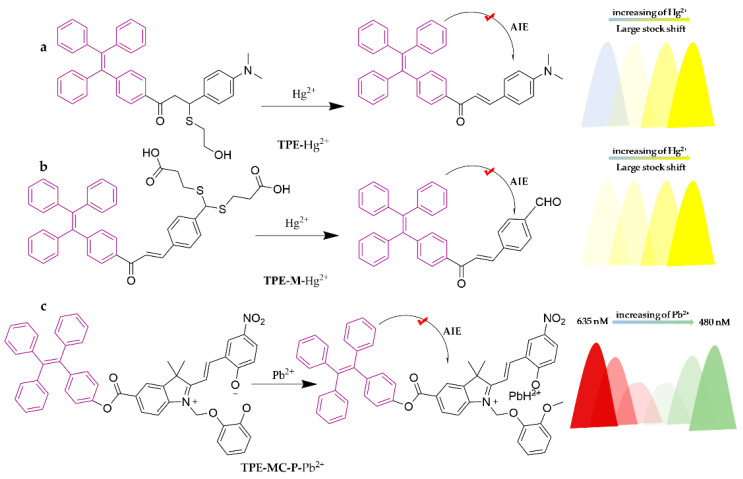
The structures of probes ((**a**) is **TPE**, (**b**) is **TPE-M,** (**c**) is **TPE-MC-P**).

The TPE molecular architecture features a propeller-shaped structure with four benzene rings connected to the middle vinyl group by C-C single bonds, and the benzene rings rotate and vibrate freely around the single bond (Scheme 2g). The phenyl ring remains in a low fluorescence state owing to the energy consumption caused by rotational vibrations when it is in solution. However, when the molecule occupies an aggregated state, such as a concentrated solution or a solid state, the non-radiative relaxation will be suppressed due to intermolecular interactions. These interactions inhibit the energy-consuming motion of the benzene ring molecules, eventually bringing out the emission of excited-state energy through fluorescence. TPE is a well-known luminescent material with AIE properties. However, TPE aggregates have a limited emission wavelength, which restricts further investigation. To overcome this limitation, a TPE-based fluorescent probe called **TPE-Hg** was synthesized by extending the conjugation of the TPE fluorophore with a hydroxyethyl sulfide portion [116]. This probe exhibits a large Stokes shift (203 nm), strong anti-interference ability, and high sensitivity and specificity in detecting Hg^2+^. In a THF/HEPES system (20 mM, pH = 7.3) (1:9, *v*/*v*), the probe undergoes a color change from green to yellow, with a detection limit as low as 7.548 × 10^−7^ M. It has been successfully utilized for the detection of Hg^2+^ in seafood and tea, with reliable recoveries (89.88–105.06%) and reasonable relative standard deviations (2.26–9.71%). The test strips obtained on the basis of **TPE-Hg** also exhibit rapid detection properties. Another selective fluorescent probe for Hg^2+^, **TPE-M**, was developed via incorporating a dithiocarbonyl group (3-mercaptopropionic acid) [117]. Reaction of **TPE-M** with Hg^2+^ in the MeOH/PBS (20 mM, pH = 7.4) (3:7, *v*/*v*) buffer system leads to the release of an AIE active precursor, which results in significant fluorescence enhancement. The detection limit is 4.16 × 10^−6^ M and it has been successfully implemented in real food samples such as shrimp, crab, and tea with good recoveries (93.47–105.4%). A new ratiometric fluorescent probe to recognize Pb^2+^, **TPE-MC-P**, was designed and synthesized by introducing a spiro-pyran (SP) unit through the phototransformation technique [118]. The probe identified Pb^2+^ in the THF/H_2_O (1:9, *v*/*v*) system, followed by a burst of red fluorescence of the MC unit (E_m_ = 635 nm) and an enhancement of the blue-green fluorescence of the TPE unit (E_m_ = 480 nm) in **TPE-MC-P**. By monitoring the fluorescence variation behavior of the probe with increasing concentrations of Pb^2+^, a new high-efficiency and high-accuracy detection method with a detection limit of 0.27 μM was established and successfully applied to potable water detection. The specific structures of the probes are shown in Figure 17.

### 3.8. Thiophene-Based Fluorescent Probes

**Figure 18:**
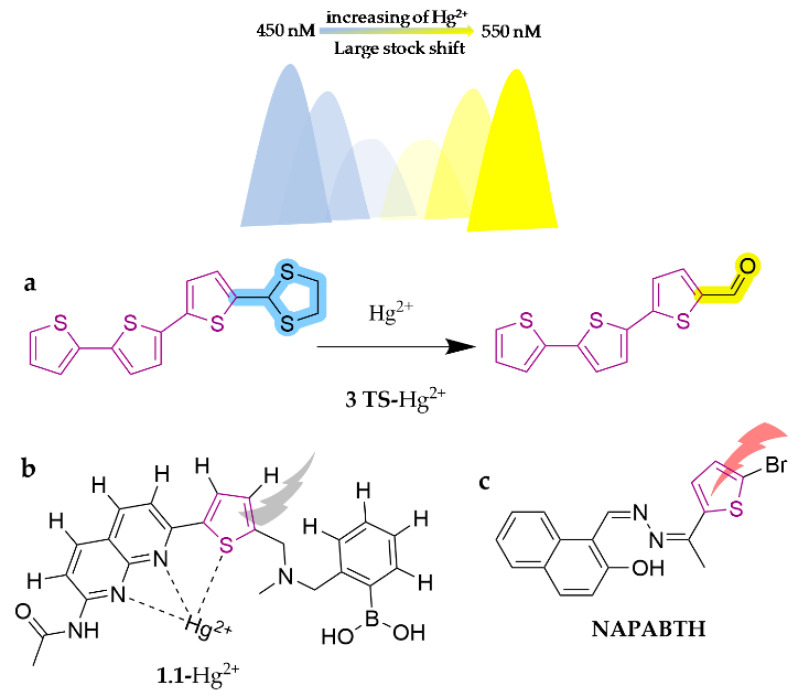
The structures of probes ((**a**) is **3 TS**, (**b**) is **1.1**, (**c**) is **NAPABTH**).

Thiophene is widely employed as the linking part of compounds due to its simplicity of structure, facile modification, and singular property (Scheme 2h). A new simple and efficient oligothiophene-based colorimetric and ratiometric fluorescent probe, **3 TS**, has been developed for the highly sensitive and fast detection of Hg^2+^ in water, soil, and seafood [119]. It works in the EtOH/H_2_O system (1:1, *v*/*v*) via the reaction of thioacetals, which elicits a significant change in the color of the probe from colorless to yellow, a 100 nm red shift of the spectrum, and intensive fluorescence enhancement. The concentration of Hg^2+^ can be determined by observing the variation in the fluorescence intensity ratio at 550 and 450 nm under Ex.360 nm, with a detection limit of up to 1.03 × 10^−8^ M. It has been successfully implemented in detecting various water samples, agricultural soils, and aquatic products. Another probe, **1.1**, was reported as a novel fluorescent chemosensor based on naphthyridine-boronic acid-thiophene derivatives [120]. This probe displays a highly selective fluorescence interruption for Hg^2+^ in the MeOH/H_2_O system by means of the PET mechanism. Interestingly, the sensitivity of **1.1** toward Hg^2+^ is enhanced at least 7-fold in the presence of physiological concentrations of D-fructose, which may be attributed to the synergistic binding of D-fructose along with mercury ions to the sensor. This is the first proposed D-fructose-mercury chemosensor until now, which facilitates the application of the probe in the food industry, for instance to detect mercury contamination in high-fructose corn syrup. Recently, hydrazones have garnered significant attention in chemistry due to the presence of reactive amino-type nitrogen in the imine core unit. Attributable to their similarities with carbonyl-containing compounds, hydrazones can participate in a huge variety of typically synthetic reactions, including free radical reactions, cyclo-addition reactions, and transition metal-based reactions [121]. They show an extensive array of intriguing organic activities and pharmacological behaviors. Based on these properties, **NAPABTH** was designed and synthesized using thiophene-functionalized hydrazone as a chemical probe for specifically sensing Pb^2+^ in the DMSO system [122]. It evokes a light yellow to pink dye color with a very low detection limit (1.06 ppm) via the ICT and LMCT processes. The specific structures of the probes are shown in Figure 18.

### 3.9. Naphthoimide-Based Fluorescent Probes

**Figure 19:**
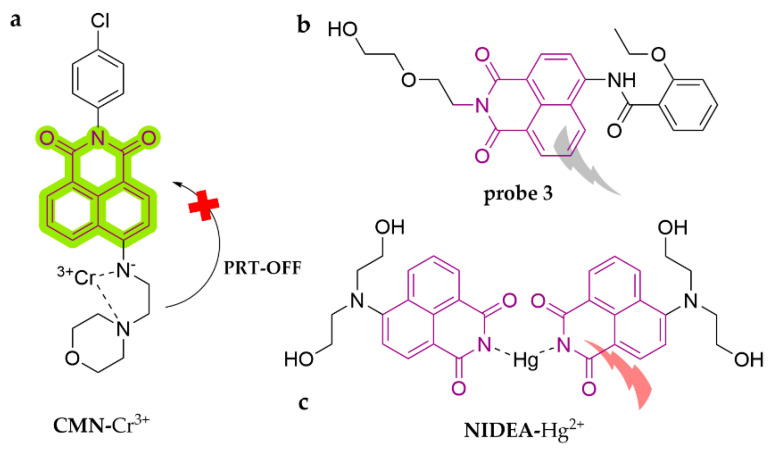
The structures of probes ((**a**) is **CMN**, (**b**) is **probe 3**, (**c**) is **NIDEA**).

With strong yellow-green fluorescence, high fluorescence quantum yields, and easy modification, naphthalimide and its derivatives have been widely used as fluorophores to develop many fluorescent dyes (Scheme 2i). As shown in Figure 19, an effective morpholine-type naphthalimide probe, N-p-chlorophenyl-4-(2-aminoethyl)morpholine-1,8-naphthalimide (**CMN**), has been developed as a lysosome-targeted fluorometric sensor for Cr^3+^ [123]. It functions in MeOH following a marked color change (yellowish color darkening), with a detection limit of 0.68 μM. Due to the N-atom of morpholine being directly involved in complex formation, **CMN** emits a fluorescence response through the inhibition of PET. Significantly, cellular confocal microscopic research indicated that the introduction of the lysosome-targeted group of the morpholine moiety realized the capability of imaging lysosomal trivalent metal ions in living cells for the first time. In another study, **probe 3** was designed as a quinoline-modified naphthalimide fluorescent probe [124]. The probe is capable of Cr^6+^ recognition in the ACN/HEPES (10 mM, pH = 7.4) (1:9, *v*/*v*) system, with a rapid fluorescence burst via the internal filtration effect. The burst constant is 7.99 × 10^3^ M^−1^ and the detection limit is 1.15 μM. The results of spiked recovery experiments in real water samples showed positive outcomes. A simple water-soluble naphthalimide-derived fluorescent dye with AIEE characteristics was reasonably constructed based on the twisted intramolecular charge transfer (TICT) mechanism. **NIDEA** was synthesized to demonstrate this mechanism [125]. It reacts well with Hg^2+^ in HEPES buffer (10 mM, pH = 7.4), specifically binding to the N-unsubstituted naphthalimide group to form a classical “imide-mercury-imide” structure. Moreover, the introduction of the diethanolamine moiety enhances the water solubility of the probe and allows for the feature of AIEE, following switching on of fluorescence, with a detection limit of 46.7 nM. It works well in real water samples.

### 3.10. Other Scaffolds

In summary, the field of fluorescent probes has witnessed significant advancements in the development of various scaffolds. These probes exhibit excellent selectivity, sensitivity, and real-time detection capabilities for heavy metals in food. However, a major drawback is that many of these probes suffer from aggregate-induced quenching (ACQ), resulting in significant interference from either the probes themselves or the background, thereby hindering their practical applications. Moreover, the limited specificity of probes remains a challenge for their application in the quantitative analysis of heavy metals in food. In particular, metal ions from the same homologous main group of the periodic table often exhibit indistinguishable responses to the same signal, leading to a lack of selectivity among diverse fluorescent probes owing to their similar electronic configurations, coordination numbers, and chemical properties. Thus, overcoming the contradictions between the above chemical properties and sensing mechanisms will be significant to developing new fluorescent probes.

#### 3.10.1. Fluorescent Probes to Detect Cd^2+^

**Figure 20:**
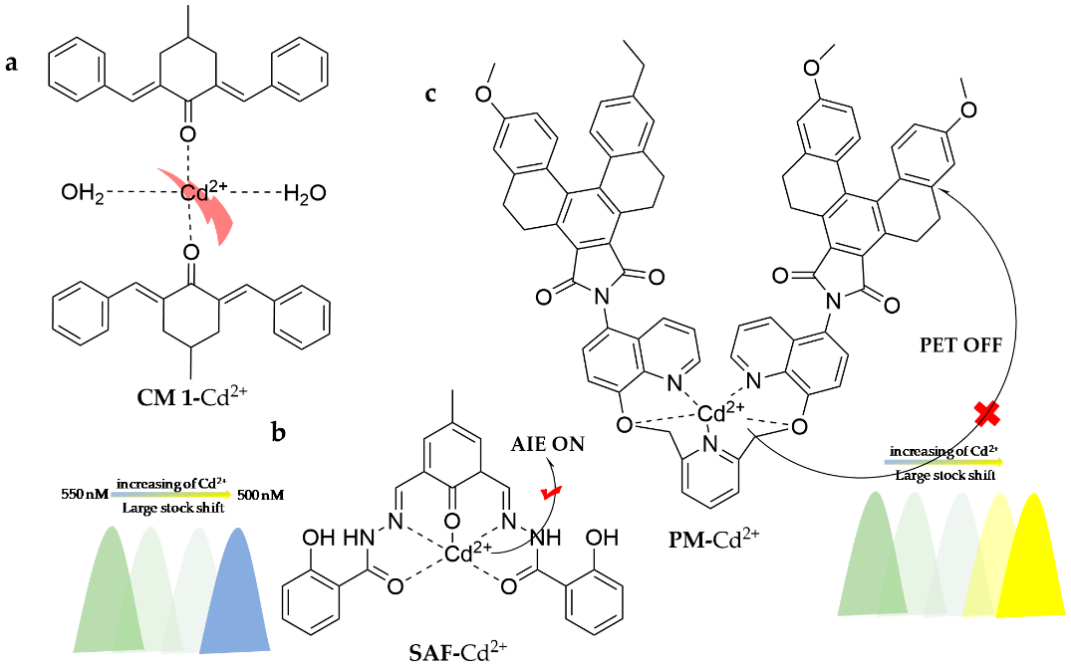
The structures of probes ((**a**) is **CM 1**, (**b**) is **SAF**, (**c**) is **PM**).

As shown in Figure 20, a novel optical chemosensor, **CM 1** (2,6-di((E)-benzylidene)-4-methylcyclohexan-1), was designed to specifically detect Cd^2+^ [126]. It works well in aqueous media, initiates fluorescence turn-on with a detection limit of 19.25 nM, and has been successfully employed in the detection of real water samples. A facile AIE fluorescent probe, **SAF**, was developed to recognize Cd^2+^ in ACN aqueous solution (95:5, *v*/*v*),exhibiting a large Stokes shift of 202 nm [127]. The probe presents high selectivity by inhibiting the ESIPT process, leading to an observable blue shift and significantly enhanced fluorescence, with the color changing from green to cyan. The recognition of Cd^2+^ is completely free from the interference of Zn^2+^. The detection limit is 1.5 × 10^−7^ M and it has successfully performed in real water samples. Throughout connecting a tetrahydro-[5]spiroene derivative fluorescent dye with 6-bis((quinolin-8-yloxy)methyl)pyridine, a novel fluorescent sensor, **PM**, was designed and synthesized for detection of Cd^2+^ [128]. The modification of the tetrahydro-[5]spiroene dye in the probe offers a strong fluorescence signal in the visible region, high fluorescence quantum yield, and a large Stokes shift, which contributes tremendously to the sensitivity of the sensor. **PM** works well in the H_2_O/dioxane (1/19, *v*/*v*) system, exhibiting turn-on fluorescence with a significantly large Stokes shift at 163 nm. The limit of detection is 53 nM and it has been successfully utilized towards Cd^2+^ detection in commercially available foods, including drinking water and rice.

#### 3.10.2. Fluorescent Probes to Detect Cr^3+^

**Figure 21:**
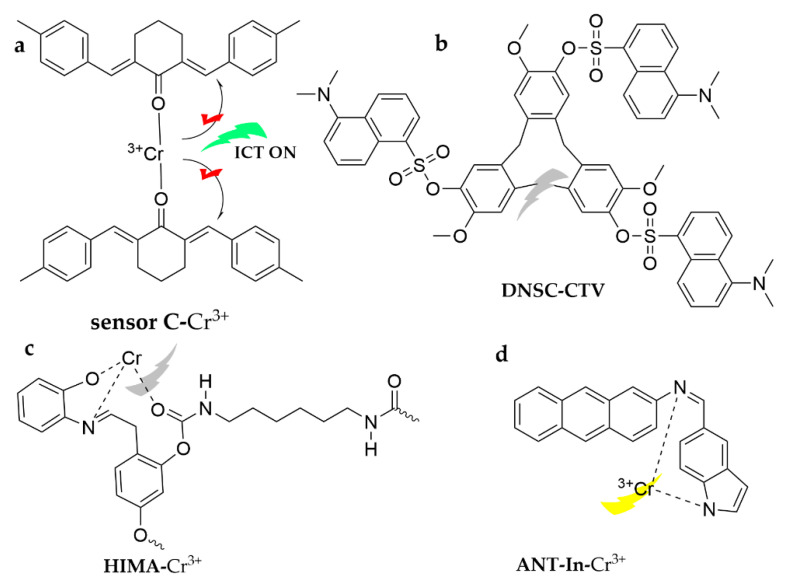
The structures of probes ((**a–d**) are **sensor C**, **DNSC-CTV**, **HIMA** and **ANT-In,** respectively).

As shown in Figure 21, the ligand 2,6-bis(E)-4-methylbenzylidine)-cyclohexan-1-one has been synthesized as a fluorescence-on probe, **sensor C**, for the trace level detection of Cr^3+^ [129]. The probe selectively responds to Cr^3+^ in ACN medium based on the ICT mechanism, turning on fluorescence with a detection limit of 2.3 × 10^−9^ M. The probe has been successfully applied to real water samples. Dansulfonyl chloride has drawn widespread attention as a fluorophore owing to its strong fluorescence, long emission wavelength (400–600 nm), large Stokes shift (330–350 nm), and easy modification. Based on these considerations, **DNSC-CTV** was published as a novel cyclotrisveratrole-derived chemosensor modified with dansyl chloride [130]. It works well for Cr^3+^ recognition in ACN. A novel poly(methylene-carbamate) chemical sensor, **HIMA**, was prepared in a two-step reaction using hexamethylene diisocyanate, 2,4-dihydroxy benzaldehyde, and 2-aminopheno, which could detect Cr^3+^ cations in different solutions [131]. It shows specific sensitivity in DMF/H_2_O (1:2, *v*/*v*), with a detection limit of 7.98 × 10^−7^ M, and has been successfully employed in different potable water samples. A novel fluorescent probe, **ANT-In**, was investigated based on anthracene and indole as the structural units [132]. It has been demonstrated that aminoanthracene-based probes exhibit structural simplicity, high quantum yield, chemical stability, and facilitate chemical modifications. Thus, the probe can identify Cr^3+^ in a highly sensitive and selective manner in the ACN/HEPES buffer (7:3, *v*/*v*, pH = 7) system via hydrolysis of the C=N bond, turning on fluorescence in less than one minute, with a low detection limit of 0.2 µM, and has been successfully implemented in potable water detection.

#### 3.10.3. Fluorescent Probes to Detect Hg^2+^

**Figure 22:**
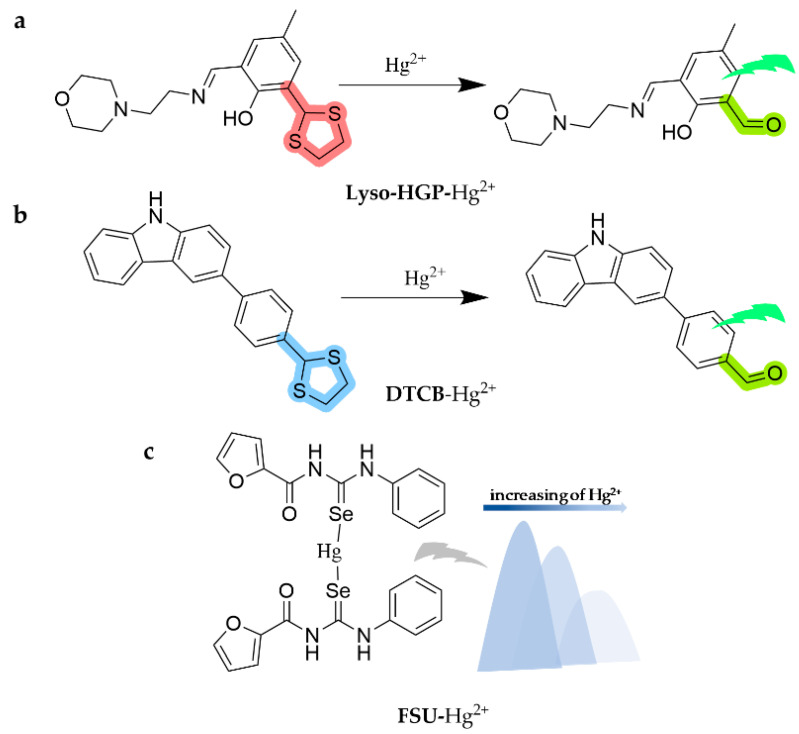
The structures of probes ((**a**) is **Lyso-HGP**, (**b**) is **DTCB**, and (**c**) is **FSU**).

As shown in Figure 22, a lysosome-targetable fluorescence sensor, **Lyso-HGP**, was designed and synthesized based on 4-methyl-2,6-diformylphenol as a fluorophore [133]. The sensor is capable of acquiring Hg^2+^ in a HEPES buffered solution (10 mM, pH = 7.0, DMSO 1%), forming a particularly fluorescent formyl-functionalized component (**Lyso-HGP**-CHO) and enhancing turn-on fluorescence by 180-fold just after 10 min. The detection limit is as low as 6.82 nM and it has been successfully applied to different drinking water assays. Also, the sensor has been applied to monitor the subcellular distribution of Hg^2+^ specifically localized in the lysosomal compartment in MCF7 human breast cancer cells by fluorescence microscopy. A new carbazole-based fluorescent probe (**DTCB**) was proposed using the theory of mercury-initiated thiolate deprotection response [134]. The probe identifies Hg^2+^-induced thioacetal in the THF/H_2_O mixture, evolving deep blue fluorescence towards green fluorescence and greatly strengthening the fluorescence intensity. It presents an excellent positive linear relationship with the concentration of Hg^2+^ at 480 nm, with a detection limit as low as 2.05 × 10^−7^ M. Moreover, the researchers extended the use of the probe in real water samples and prepared test strips. A novel selenium-based compound probe, **FSU,** based on N-(phenylcarbamoselenoyl) furan-2-carboxamide, was presented for the optical and fluorimetric detection of Hg [135]. The sensor is able to recognize Hg^2+^ in the DMSO/H_2_O (95:5, *v*/*v*) system, indicating a fluorescence burst with a detection limit of 7.35 × 10^−7^ M, in which selenium behaves as a magnet for mercury based on ICT.

#### 3.10.4. Fluorescent Probes to Detect Pb^2+^

**Figure 23:**
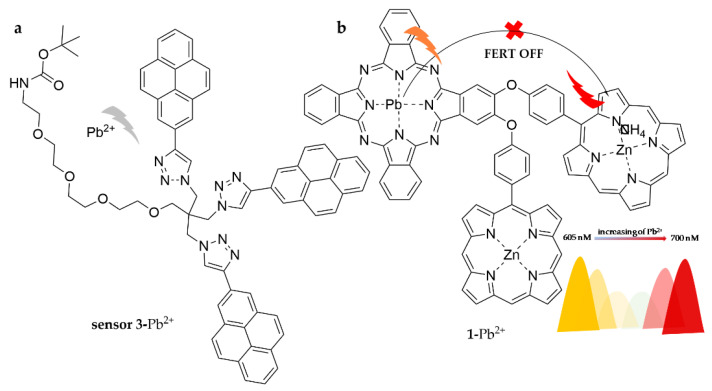
The structures of probes ((**a**) is **sensor 3** and (**b**) is **1**).

Pyrene derivatives are commonly encountered as chemosensors. In contrast to general pyrene derivatives, pyrene derivatives functionalized at the 2-position by a one-step iridium-catalyzed reaction of B2pin2 exhibit a prolonged fluorescence lifetime, which facilitates enhanced response and improved sensitivity. According to this, V. Merz et al. [136] designed and synthesized a molecular sensor, **sensor 3**, composed of pyrene and tetraethylene glycol together. The sensor could distinguish Pb^2+^ in ACN with a detection limit of 6 × 10^−7^ M and has been successfully operated in water samples. Recently, porphyrins and phthalocyanines have been frequently employed to construct ratiometric metal ion sensors in view of their tunable photophysical properties and metal ion-binding characteristics. For example, the phthalocyanine fraction (**H_2_Pc**) could selectively bind Pb^2+^, showing fluorescence quenching and inhibiting the intramolecular **FRET** process, thus turning on the fluorescence of the porphyrin fraction (**ZnPor**) [137]. In this way, **1** was constructed as a novel phthalocyanine-porphyrin triplet [H_2_Pc-β-(ZnPor)_2_] ratiometric fluorescent probe [138]. The probe is utilized in the THF/MeOH (4:1, *v*/*v*) system to identify Pb^2+^. It is possible to achieve detection of Pb^2+^ with a limit of detection of 4.1 nM through monitoring the fluorescence behaviors at 605 nm (**ZnPor** turn-on) and 700 nm (**H_2_Pc** turn-off) corresponding with increasing concentrations of Pb^2+^ under Ex.420 nm. The structures of the probes are shown in Figure 23.

Overall, researchers have developed numerous novel fluorophores or recognition groups in recent years that have broadened the scope of applications for fluorescent probe technology in heavy metal detection in food. Table 2 lists the analytes, detection limits, working solutions, usage, and references for all probes presented in this review. They all exhibited high detection capabilities for various heavy metals in food; however, there remain some challenges and limitations. For example, the heavy metals identified using the fluorescent probes reported so far almost present only in the form of inorganic ions. In practice, heavy metals are also found in organic form in food, such as mercury [139]. Hence, it is still necessary to develop further fluorescent materials which enable the rapid, accurate, and nondestructive detection of organic forms of heavy metals. Then, summarizing the above discussion, it is clear that most of the available fluorescent probe-based assays work in solution media and depend on large laboratory instruments, so developing mobile instruments such as solid-loaded test kits or test strips for daily lives is urgently needed, which requires strong stability, interference resistance, photobleaching capability, simpler operation systems, etc. for the developed fluorescent probes. Although few researchers have integrated fluorescent probes with nanomaterials to overcome the limitations mentioned above, the designed nanosensors may exhibit nonspecific incorporations within complex food matrices, leading to potential false approvals [140], which considerably restricts their wider practical application in the food industry. Consequently, exploiting extra fluorescent materials is required for improved probe utilization. This is challenging, yet we firmly acknowledge that this area of investigation is going to evolve rapidly via more research. We hope this review will facilitate developments in fluorescent probe detection of heavy metal ions in food and ultimately safeguard food safety and public health.

## 4. Conclusions and Outlook

In the past decades, heavy metal accumulation in food has emerged as a major problem contributing to food safety issues. Rapid and accurate determination of heavy metals in food draws much more critical attention in safeguarding food safety. As an emerging photochemical assay technique possessing the advantages of sensitivity, convenience, accuracy, cost, and reliability, the fluorescence assay is becoming more and more practical for determining hazards in the food industry in recent years, especially toxic heavy metals, but only a few summarizing articles are available in this field. Hence, this review systematically presents the recent strides in the construction and potential applications of novel fluorescent probes for food heavy metal detection in the past five years, which are categorized according to fluorophores and newly emerging sensing cores. All endeavors could contribute to broadening the prospects of fluorescent materials in the establishment of rational assays for food safety.

## Figures and Tables

**Table 2:** Applications of fluorescent materials for heavy metal detection in food.

Probe	Analytes	LOD	Working Solution	Application	Ref.
R1	Pb^2+^	2.7 × 10^−9^ M	DMSO	seafood	[40]
REHBA	Pb^2+^	0.73 μM	Tris-HCl (10 mM, pH = 7.0)	water	[41]
3	Hg^2+^	15.80 nM	MeOH/HEPES (1:9, *v*/*v*, pH = 7.4)	cell imaging	[42]
HL-CHO	Cr^3+^				
p-RPT	Hg^2+^	1.2 × 10^−8^ M	THF/H_2_O (3:2, *v*/*v*)	water	[43]
d114	Hg^2+^	8.6 nM	MeOH/H_2_O (1:1, *v*/*v*)	drinking water	[44]
FO_511_	Hg^2+^	92.7 nM	HEPES (10 mM, pH = 7.2)	cell imaging	[45]
RBLY	Hg^2+^	0.34 μM	EtOH/H_2_O (1:5, *v*/*v*)	water	[46]
R6GH	Pb^2+^	0.02 μM	THF/H_2_O (1:1, *v*/*v*)	seafood	[47]
NA-RhB	Pb^2+^	0.00001 g·L^−1^		water	[48]
FP	Pb^2+^	0.42 μM	EtOH/H_2_O (99:1, *v*/*v*)	tap water	[49]
	Cd^2+^	0.53 μM			
RhBQ	Cr^3+^	2.12 × 10^−8^ M	ACN/H_2_O (9:1, *v*/*v*)	cell imaging	[50]
RFC	Cr^3+^	0.0052 ppm	MeOH/H_2_O (99:1, *v*/*v*)	cell imaging	[51]
1 O	Cd^2+^		ACN		[52]
Receptor	Cr^3+^	3.92 μM	H_2_O	zebrafish	[53]
C6	Cr^3+^	13.3 µM	ACN	tap water	[54]
PBD	Cr^3+^	0.32 μM	EtOH/H_2_O (1:1, *v*/*v*)	water, soil	[55]
	Hg^2+^	1.93 μM			
P	Cr^3+^	9.82 × 10^−9^ M	DMF/H_2_O (9:1, *v*/*v*)	water	[56]
SB2	Cr^3+^	0.5 µM	MeOH/H_2_O (3:1, *v*/*v*)	soil	[57]
HMA	Cr^3+^	5.63 × 10^−7^ M	DMSO/H_2_O (9:1, *v*/*v*)	water	[58]
NHT	Cr^3+^	41 nM	HEPES (0.2 mM, pH = 7.2)	cell imaging	[59]
L1	Cr^3+^	1.12 × 10^−7^ M	ACN/H_2_O (1:1, *v*/*v*)	water	[60]
L2	Cr^3+^	7.73 × 10^−7^ M			
1 O	Cd^2+^	5.74 × 10^−7^ M	THF	water	[62]
PIS	Cd^2+^	2.10 × 10^−8^ M	ACN/HEPES (10 mM, pH = 7.4) (1:4, *v*/*v*)	zebrafish	[61]
PMPA	Cd^2+^	0.12 mM	ACN	water	[63]
DBTBH	Pb^2+^	4.49 × 10^−8^ M	THF/Tris-HCl (10 mM, 1 mM KI, pH = 7.4) (1:9, *v*/*v*)	water	[64]
BSBBT	Pb^2+^	2.23 × 10^−6^ M	DMSO/H_2_O (3:7, *v*/*v*)		[65]
L	Pb^2+^	9 × 10^−7^ M	MeOH/Tris (1:1, *v*/*v*)	water	[66]
probe 1	Cd^2+^	0.055 μM	ACN	water, bean sprouts	[67]
QTPY	Cd^2+^	3.5 × 10^−8^ M	DMF/H_2_O (4:6, *v*/*v*)		[68]
L	Cd^2+^	14.8 nM	ACN/H_2_O (8:2, *v*/*v*)	water	[69]
DDTQ	Cd^2+^	126 nM	H_2_O	cell imaging	[70]
Pb(II)-IIP	Pb^2+^	2.1 μg∙L^−1^	H_2_O	water	[71]
QMC	Pb^2+^	13 μM	ACN/H_2_O (1:1, *v*/*v*)	milk, wine	[72]
IL [HDQ] [P66614]	Hg^2+^	0.8 nM	EtOH	water, seafood	[73]
DAC-Hg	Hg^2+^	5.0 nM	PBS	water, seafood, soil	[74]
HCDC	Hg^2+^	0.3 nM	HEPES (5 M, pH = 7.4)	water	[75]
L	Hg^2+^	5.15 nM	bis-tris (10^−2^ M, pH = 7.0, 0.5% DMSO)	water	[76]
1a	Cd^2+^	5.84 × 10^−8^ M	HEPES (20 mM, ACN/H_2_O, 3:7, *v*/*v*, pH = 7.0)		[77]
1	Cd^2+^	0.114 μM	THF/H_2_O (1:1, *v*/*v*)		[78]
C1-C3	Pb^2+^	0.009–0.0015	ACN		[79]
NIS	Cd^2+^	3.87 × 10^−7^ M	HEPES (EtOH/H_2_O = 9:1, *v*/*v*, pH = 7.4)	zebrafish	[80]
BPC	Cd^2+^	1.05 × 10^−8^ M	ACN/Tris-HCl (3:2, *v*/*v*, pH = 7.4)	water	[81]
[PTZ-SB][Br]	Cd^2+^	3.8 × 10^−7^ M	THF/H_2_O (1:9, *v*/*v*)	water	[82]
IHL	Cd^2+^	0.4 × 10^−10^ M	DMSO/H_2_O (9:1, *v*/*v*)	zebrafish	[83]
L1	Hg^2+^	7.6 × 10^−6^ M	ACN/H_2_O (1:1, *v*/*v*)		[84]
	Pb^2+^	8.5 × 10^−6^ M	ACN		
L2	Hg^2+^	6.7 × 10^−6^ M	ACN/H_2_O (1:1, *v*/*v*)		
	Pb^2+^	2.5 × 10^−6^ M	ACN		
compound 1	Hg^2+^	45.76 nM	DMF/PBS (1:4, *v*/*v*, pH = 7.4)	water	[85]
L	Cd^2+^	0.03 ppm	ACN/H_2_O (4:1, *v*/*v*)		[86]
NBDT	Cr^3+^	0.041 μM	DMSO/H_2_O (9:2, *v*/*v*)	zebrafish	[87]
CY	Hg^2+^	1.61 × 10^−7^ M	DMSO/H_2_O (7:3, *v*/*v*)	water	[88]
BTS	Pb^2+^	0.67 µM	DMSO/H_2_O (1:4, *v*/*v*)	water	[89]
NBD-MPA	Hg^2+^	9.2 × 10^−7^ M	ACN/HEPES (1:9, *v*/*v*)	water, soil, green tea, seafood	[90]
PY	Cd^2+^	0.09 μM	MeOH (pH = 7, PBS)	water	[91]
BPP		0.02 nM	DMSO/H_2_O (9:1, *v*/*v*)	zebrafish	[92]
ADMPA		29.3 nM	DMF/H_2_O (3:7, *v*/*v*)	water	[93]
3	Pb^2+^	2.31 × 10^−6^ M	ACN		[94]
NT	Cd^2+^		ACN		[95]
H_2_L	Cd^2+^	2.67 × 10^−8^ M	MeOH	food	[96]
TND	Pb^2+^	4.7 nM	ACN/H_2_O (1:1, *v*/*v*)	water	[97]
probe 1	Pb^2+^	1.65 nM	ACN/EtOH/HEPES (1:1:2, *v*/*v*/v, pH = 7.0)	water	[98]
TPA-TSC	Hg^2+^	0.14 nM	ACN/PBS (1:1, *v*/*v*, pH = 7.4)	sea bass, water, swordfish	[99]
TPE-Hg	Hg^2+^	7.548×10^−7^ M	THF/HEPES (20 mM, pH = 7.3) (1:9, *v*/*v*)	green tea, seafood	[100]
TPE-M	Hg^2+^	4.16×10^−6^ M	MeOH/PBS (20 mM, pH = 7.4) (3:7, *v*/*v*)	green tea, seafood	[101]
TPE-MC-P	Pb^2+^	0.27 μM	THF/H_2_O (1:9, *v*/*v*)	water	[102]
3 TS	Hg^2+^	1.03 × 10^−8^ M	EtOH/H_2_O (1:1, *v*/*v*)	water, soil, seafood	[103]
1.1	Hg^2+^		MeOH/H_2_O	high-fructose corn syrup	[104]
NAPABTH	Pb^2+^	1.06 ppm	DMSO		[105]
CMN	Cr^3+^	0.68 μM	MeOH		[106]
probe 3	Cr^6+^	1.15 μM	ACN/HEPES (10 mM, pH = 7.4) (1:9, *v*/*v*)	water	[107]
NIDEA	Hg^2+^	46.7 nM	HEPES (10 mM, pH = 7.4)	water	[108]
CM 1	Cd^2+^	19.25 nM	H_2_O	water	[109]
SAF		1.5 × 10^−7^ M	ACN/H_2_O (95:5, *v*/*v*)	water	[110]
PM	Cd^2+^	53 nM	H_2_O/dioxane (1/19, *v*/*v*)	water, rice	[111]
sensor C	Cr^3+^	2.3 × 10^−9^ M	ACN	water	[112]
DNSC-CTV			ACN		[113]
HIMA		7.98 × 10^−7^ M	DMF/H_2_O (1:2, *v*/*v*)	drinking water	[114]
ANT-In		0.2 µM	ACN/HEPES (7:3, *v*/*v*, pH = 7)	drinking water	[115]
Lyso-HGP	Hg^2+^	6.82 nM	HEPES (10 mM, pH = 7.0, 1%DMSO)	water	[116]
DTCB		2.05 × 10^−7^ M	THF/H_2_O	water	[117]
FSU		7.35 × 10^−7^ M	DMSO/H_2_O (95:5, *v*/*v*)		[118]
sensor 3	Pb^2+^	6 × 10^−7^ M	ACN	water	[119]
1		4.1 nM	THF/MeOH (4:1, *v*/*v*)		[121]

## Data Availability

The data that support the findings of this study are available from the corresponding author upon reasonable request.
